# Effects of irrelevant unintelligible and intelligible background speech on spoken language production

**DOI:** 10.1177/17470218231219971

**Published:** 2024-01-21

**Authors:** Jieying He, Candice Frances, Ava Creemers, Laurel Brehm

**Affiliations:** 1 Max Planck Institute for Psycholinguistics, Nijmegen, The Netherlands; 2 International Max Planck Research School for Language Sciences, Nijmegen, The Netherlands; 3 Department of Linguistics, University of California, Santa Barbara, Santa Barbara, CA, USA

**Keywords:** Irrelevant speech effect, name agreement, speech production

## Abstract

Earlier work has explored spoken word production during irrelevant background speech such as intelligible and unintelligible word lists. The present study compared how different types of irrelevant background speech (word lists vs. sentences) influenced spoken word production relative to a quiet control condition, and whether the influence depended on the intelligibility of the background speech. Experiment 1 presented native Dutch speakers with Chinese word lists and sentences. Experiment 2 presented a similar group with Dutch word lists and sentences. In both experiments, the lexical selection demands in speech production were manipulated by varying name agreement (high vs. low) of the to-be-named pictures. Results showed that background speech, regardless of its intelligibility, disrupted spoken word production relative to a quiet condition, but no effects of word lists versus sentences in either language were found. Moreover, the disruption by intelligible background speech compared with the quiet condition was eliminated when planning low name agreement pictures. These findings suggest that any speech, even unintelligible speech, interferes with production, which implies that the disruption of spoken word production is mainly phonological in nature. The disruption by intelligible background speech can be reduced or eliminated via top–down attentional engagement.

## Introduction

Much of daily conversation, which requires both speech comprehension and production, occurs in the presence of irrelevant external auditory stimulation, including noise from nearby traffic or construction, a television broadcasting in the background, or a colleague talking on the phone. Extensive work has shown that background noise, music, and speech all have detrimental effects on spoken language comprehension (e.g., Eckert et al., 2016). However, very few studies have investigated how speakers plan their speech in the presence of irrelevant background noise, especially irrelevant background speech (e.g., Fargier & Laganaro, 2016, 2019; He, Meyer & Brehm, 2021). Understanding speech production in non-verbal and verbal sources of noise advances our understanding of how speakers cope with auditory disruption when planning their speech. The present study thus investigated how different types of irrelevant background speech (word lists and sentences) influenced spoken word production with varying lexical selection demands, and whether the influence was modulated by the difficulty of speech production.

### One irrelevant speech effect, two relevant theories

Previous studies have found that speech and non-speech sounds disrupt cognitive tasks such as serial recall (e.g., Parmentier & Beaman, 2015; Röer et al., 2014, 2015; Schlittmeier et al., 2012) and reading (e.g., Cauchard et al., 2012; Hyönä & Ekholm, 2016; Yan et al., 2018), even when they are irrelevant for the task and can be ignored. This is referred to as the *irrelevant speech effect* (or *irrelevant sound effect*; Colle & Welsh, 1976; Jones & Morris, 1992). One major account for the irrelevant speech effect is the involvement of shared mechanisms or representations in both tasks; this is known as the *domain-specific interference-by-similarity account* (e.g., Jones et al., 1993; Martin et al., 1988; Salamé & Baddeley, 1982, 1989). This was first proposed to explain the changing-state effect in serial recall where distractor sequences like A B C D E F G H disrupt more than A A A A A A A A (Hughes, 2014; Hughes et al., 2007; Jones et al., 1993; Jones & Morris, 1992). The effect has been attributed to conflict driven by automatic processing of the irrelevant auditory distractors’ order (*interference-by-process account*; e.g., Hughes, 2014; Jones et al., 1993). This interference-by-similarity account resembles the crosstalk account for dual-task processing based on neural resources (Pashler, 1994; *outcome conflict*: Navon & Miller, 1987), claiming that shared or similar representations or processes cause interference in task performance.

Two hypotheses attribute the irrelevant speech effect to different sources that are both important to consider for the effect of background speech on speech production. The *phonological disruption view* (Salamé & Baddeley, 1982, 1989) hypothesises that the irrelevant speech effect results from the similarity in content of phonological codes (e.g., reading and irrelevant background speech), which are both buffered in a phonological memory store (a component of the phonological loop; Baddeley, 2000, 2003). This view predicts that disruption in speaking should occur from the presence of irrelevant background speech, regardless of its content. By contrast, the *semantic disruption view* (Martin et al., 1988) attributes the effect to the shared use of semantic processing (e.g., English reading is disrupted more by English-intelligible- than Russian-unintelligible-background speech). This view predicts that disruption in speaking should be produced by intelligible meaningful speech because meaningless speech does not recruit semantic processing.

In contrast to the domain-specific interference-by-similarity, the *domain-general attention capture* account posits that irrelevant speech or sound disrupts focal task performance by diverting attention away from the task (Buchner et al., 2004; Cowan, 1995; Elliott & Briganti, 2012; Röer et al., 2013, 2015). When the focus of attention is captured by task-irrelevant sounds, fewer attentional resources are available and task performance is impaired. The attention capture theory has some support in how irrelevant background speech interferes with serial recall performance (e.g., Buchner et al., 2004; Cowan, 1995; Elliott & Briganti, 2012; Röer et al., 2013, 2015) and reading (e.g., Hyönä & Ekholm, 2016). This attention capture account is compatible with the capacity limitation account for dual-task processing (Pashler, 1994; Ruthruff et al., 2003), which states that the amount of attentional resources available to focal cognitive tasks determines task performance.

There is a similar divide within this domain-general attention capture view with different predictions of the effects of irrelevant background on speech production (Eimer et al., 1996). *Aspecific attention capture* occurs when a sound captures attention because of the context in which it occurs, such as the sudden onset of speech following a period of silence (Eimer et al., 1996). This view predicts that irrelevant background speech with varied context (stimulus-*aspecific* variation, e.g., pauses in speech) should interfere more with the focal task than background speech with constant context (e.g., continuous speech). Alternatively, *specific attention capture* can occur when the content of the sound diverts attention (e.g., Eimer et al., 1996; Röer et al., 2013; Wood & Cowan, 1995), which implies that the attention-diverting power is attributable to the stimulus itself (stimulus-*specific* variation). This view predicts irrelevant background speech with rich linguistic representations (e.g., full sentences) should elicit more disruption than that with less linguistic information (e.g., word lists).

### Irrelevant speech effects in spoken language production

The earlier work is nearly all conducted on language comprehension, and importantly, similar processes may or may not be relevant for speech production. Prior literature has indicated that speech production and comprehension draw upon similar processes/representations (e.g., Glaser & Düngelhoff, 1984; Kittredge & Dell, 2016; Mitterer & Ernestus, 2008; Schriefers et al., 1990), and both require attention (Cleland et al., 2006; Lien et al., 2008; Roelofs & Piai, 2011). This implies that the domain-specific interference-by-similarity (Martin et al., 1988; Salamé & Baddeley, 1982, 1989) and domain-general attention capture (Buchner et al., 2004; Cowan, 1995; Elliott & Briganti, 2012; Röer et al., 2013, 2015) mechanisms may play roles in the disruption by irrelevant background speech on speech production. However, it is also important to note that speech production and speech comprehension are also fundamentally different processes, with different goals (production = convert message to output form; comprehension = convert input form to message), and different burdens of attention. This makes it important to systematically investigate the irrelevant speech effect in language production.

Evidence from the picture–word interference (PWI) studies (Glaser & Düngelhoff, 1984; Schriefers et al., 1990) has supported the interference-by-similarity explanation. When naming a picture (e.g., DOG) with a spoken related distractor word (e.g., FOX), naming latencies and error rates increased compared with trials with an unrelated distractor (e.g., RANK; Damian & Martin, 1999; Schriefers et al., 1990). This suggests that the distractor word activated semantic representations required by the target word, interfering with spoken word production when they are related (see Roelofs, 1992, 2003), which is consistent with the semantic disruption view (Martin et al., 1988). When naming a picture (e.g., BED), a phonologically related distractor word (e.g., BEND) elicits less interference than an unrelated distractor (e.g., DUKE) (Damian & Martin, 1999; Schriefers et al., 1990). This suggests that comprehending a distractor word pre-activates phonological representations similar to the target, facilitating production when they are related. This, in turn, implies that if what is produced mismatches with what is comprehended, pre-activation of phonological/phonetic representations could also elicit interference, which is consistent with the phonological disruption view (Salamé & Baddeley, 1982, 1989).

Fargier and Laganaro (2016) investigated the roles of both interference-by-similarity and capacity limitation mechanisms by using a dual-task paradigm. Participants named pictures in three listening conditions with varying attentional demand: without distractors (low), while passively listening to distractors (medium), and during a distractor detection task (high). The auditory distractors were either tones (non-verbal stimuli) or syllables (verbal stimuli). Production latencies were longer for syllables relative to tones, and increased for tasks with higher attentional demand. These results suggest that increased representational similarity and attentional demand cause more interference on speech production performance.

To expand on earlier work on interference between single-word production and comprehension (e.g., Fargier & Laganaro, 2016; Glaser & Düngelhoff, 1984; Schriefers et al., 1990), He, Meyer & Brehm, 2021 conducted a study which mainly supports the role of interference-by-similarity in the irrelevant speech effect for speech production. In this study, Dutch speakers named sets of pictures while ignoring Dutch word lists, Chinese word lists, or eight-talker babble (i.e., language-like noise). Irrelevant background speech (Dutch and Chinese word lists) disrupted spoken word production more than eight-talker babble, and Dutch caused more disruption than Chinese word lists. This suggests that more interference on spoken word production is obtained as the representational similarity between speech production and irrelevant background speech increases, consistent with the interference-by-similarity view (Martin et al., 1988; Salamé & Baddeley, 1982, 1989). However, He, Meyer & Brehm, 2021 did not distinguish between phonological and semantic sources of disruption, which might both contribute to interference. This study also does not rule out disruption by attention capture because the irrelevant background speech varied in both aspecific context (pauses in word lists but not in eight-talker babble) and specific linguistic content (information content in word lists but not in eight-talker babble).

Furthermore, because speaking requires attention, task demands may modulate the irrelevant speech effect in language production. He, Meyer & Brehm, 2021 also manipulated the difficulty of speech production by varying name agreement (high, low) of to-be-named pictures. Name agreement is the extent to which participants agree on the name of a picture. Previous studies have found that naming a picture with high name agreement (e.g., the item called *banana*) is faster and more accurate than naming one with low name agreement (e.g., the item called *sofa* or *couch;* e.g., Alario et al., 2004; Cheng et al., 2010; Shao et al., 2014; Vitkovitch & Tyrrell, 1995). The effect is caused by both difficulty in object recognition (confusion over what the object should be called) and the demands of lexical selection (the need to select among competing lexical candidates); He, Meyer & Brehm, 2021 used stimuli designed to elicit the latter effect. Irrelevant speech effects were strongest for high name agreement pictures with low lexical selection demands, which suggests that the interference can be eliminated when speech production is more demanding. The finding is consistent with a top–down *attention engagement mechanism* (also referred to as *task engagement*; see Halin et al., 2014; Marsh et al., 2015): difficult speech production may make speakers concentrate harder and reduce processing of irrelevant background speech. This means that to study irrelevant speech effects in speech production, it is also important to consider the production demands.

### Current study

The present study was designed to explore how different types of irrelevant background speech affected spoken language production. Given that previous studies have supported the reliability of conducting speech production research online (e.g., Fairs & Strijkers, 2021; He, Meyer, Creemers, & Brehm, 2021; Stark et al., 2022; Vogt et al., 2022), we designed two web-based experiments which focused on teasing apart the variants of the interference-by-similarity and attention capture accounts. To distinguish between the semantic and phonological interference-by-similarity views, we examined disruption by unintelligible (Chinese, Experiment 1) and intelligible background speech (Dutch, Experiment 2) on Dutch spoken word production. The phonological disruption view (Salamé & Baddeley, 1982, 1989) predicts that background speech, regardless of its intelligibility, should disrupt speech production relative to a quiet condition, predicting a similar pattern of results across experiments. By contrast, the semantic disruption view (Martin et al., 1988) predicts that only intelligible background speech should interfere with speech production, predicting more disruption in Experiment 2 than Experiment 1. The predictions for each account in the present study are shown in Table 1.

**Table 1.** A summary of predictions in the present study.

Account	Predictions
Interference-by-similarity account (e.g., Jones et al., 1993; Martin et al., 1988; Salamé & Baddeley, 1982, 1989)	
Phonological disruption view (Salamé & Baddeley, 1982, 1989)	Both Chinese speech (in Exp1) and Dutch speech (in Exp2) should disrupt spoken word production relative to a quiet condition.
Semantic disruption view (Martin et al., 1988)	Chinese speech (in Exp1) should not disrupt spoken word production relative to a quiet condition, but Dutch speech (in Exp2) should.
Attention capture account (e.g., Buchner et al., 2004; Cowan, 1995; Elliott & Briganti, 2012; Röer et al., 2013, 2015)	
Aspecific attention capture view (Eimer et al., 1996)	Exp1: Chinese word lists should be more disruptive than Chinese sentences. Exp2: Dutch word lists may be more disruptive than Dutch sentences.
Specific attention capture view (Eimer et al., 1996)	Exp1: Chinese word lists should have the same disruptive potency as the sentences. Exp2: Dutch word lists may be less disruptive than Dutch sentences.
Attention engagement account (Halin et al., 2014; Marsh et al., 2015)	
Stimulus-aspecific disruption	Interference elicited by Chinese background speech (in Exp1) should not be affected by name agreement.
Stimulus-specific disruption	Interference elicited by Dutch background speech (in Exp2) should be reduced for low name agreement pictures.

In both experiments, we compared word lists containing silent pauses (e.g., *渔夫,合唱团,足球,苹果,尺子,鹿;* “*fisherman, choir, football, apple, ruler, deer*”) with sentences that form continuous speech without pauses (e.g., *鹿和尺子在苹果的左边, 并且足球和合唱团在渔夫的右边. “The deer and the ruler are to the left of the apple, and the football and the choir are to the right of the fisherman.”*). This allows us to distinguish between the two attention capture view variants (Buchner et al., 2004; Cowan, 1995; Elliott & Briganti, 2012; Röer et al., 2013, 2015). In Experiment 1, if attention capture is only caused by *aspecific* context variation (e.g., the presence/absence of pauses), Chinese word lists should elicit more interference than Chinese sentences because they contain more pauses. By contrast, if attention capture is only caused by *specific* linguistic content (e.g., semantics or syntax), Chinese word lists should cause the same disruption as the Chinese sentences because they are meaningless to our Dutch speakers. Specific and aspecific properties will also elicit similar patterns of disruption in Experiment 2, though these may be modulated by specific linguistic content because Dutch word lists and sentences differ to Dutch speakers in both semantics and syntax. We thus make relatively weak predictions under the attention capture view variants for Experiment 2. See Table 1 for more details.

In both experiments, we also investigated the role of top–down attention engagement by manipulating the name agreement (high vs. low) and therefore, lexical selection demands, of to-be-named pictures. This provides insight into whether and how speakers take top–down strategies to shield against auditory disruption when planning their speech. Following earlier work (Alario et al., 2004; Cheng et al., 2010; Shao et al., 2014; Vitkovitch & Tyrrell, 1995), we predicted that pictures with low name agreement would be named more slowly than those with high name agreement in both experiments. Interactions between the type of irrelevant background speech and name agreement also show how the irrelevant speech effects are affected by the required attentional demand of speech production. Because stimulus-aspecific disruption occurs automatically, we predicted that any interference present in Experiment 1 would not be affected by name agreement. This is because the stimulus-aspecific disruption is rooted in the automatic processing of the auditory input that escapes cognitive control (Hughes, 2014). By contrast, stimulus-specific disruption is non-automatic, which means that any disruption caused by the attention-capturing properties of intelligible background speech in Experiment 2 might be reduced for low compared with high name agreement pictures. This is because stimulus-specific disruption requires central attention that taps into cognitive control (Hughes, 2014; Marsh et al., 2018).

## Experiment 1

### Method

#### Participants

We recruited 50 native speakers of Dutch who had no experience with Chinese (45 females, *M*_age_ = 25 years, range: 20–35 years) from the participant pool at the Max Planck Institute for Psycholinguistics. Power simulations (see https://osf.io/wuafh/) showed that 50 participants and 144 items (80% of the items in the study named successfully) would provide 95% power to measure a plausibly sized condition difference of 20 ms (*SD* = 900 ms). All participants reported normal or corrected-to-normal vision and no speech or hearing problems. They signed an online informed consent form and received a payment of €6 for their participation. The study was approved by the ethics board of the Faculty of Social Sciences of Radboud University.

#### Apparatus

The experiment was implemented in FRINEX (FRamework for INteractive EXperiments; Withers, 2017), a web-based platform developed at the Max Planck Institute for Psycholinguistics. Participants used their own laptops with headphones/earphones. We restricted participation to 14-in. or larger laptops (range: 14–24 in.) with Google Chrome, Firefox, Microsoft Edge, or Brave web browsers. Each participant’s speech was recorded by a built-in voice recorder in the web browser. WebMAUS Basic was used for phonetic segmentation and transcription (https://clarin.phonetik.uni-muenchen.de/BASWebServices/interface/WebMAUSBasic). Praat (Boersma & Weenink, 2009) was then used to extract the onsets and offsets of all segmented responses.

#### Materials

##### Visual stimuli

A total of 240 pictures from He, Meyer and Brehm (2021), Experiment 2; pictures selected from the MultiPic database, Duñabeitia et al., 2018; see Supplementary Material, Table A1) were used in the present study. Of these, 120 were high name agreement pictures, all with a name agreement percentage of 100%, and 120 were low name agreement pictures, with a name agreement between 50% and 87% (*M* = 72%, *SD* = 11%). Independent *t*-tests revealed that the two sets of pictures differed significantly in name agreement, but not in any of the following psycholinguistic attributes: visual complexity, word frequency (WF), age-of-acquisition (AoA), number of phonemes, number of syllables, word prevalence, phonological neighbourhood frequency (PNF), phonological neighbourhood size (PNS), orthographic neighbourhood frequency (ONF), and orthographic neighbourhood size (ONS).

The 120 high name agreement and 120 low name agreement pictures were each divided into three subsets and paired with the two background speech conditions (Chinese word list, Chinese sentence) and a quiet control condition, meaning that each auditory condition was paired with 40 high name agreement and 40 low name agreement pictures. The three sets of pictures were matched on the 10 above-mentioned attributes, and the high and low name agreement picture sets were assigned to each auditory condition.

On each trial of the experiment, four pictures, all with high name agreement or all with low name agreement, were presented simultaneously in a 1 × 4 grid (size: 10 cm × 40 cm). The pictures per grid were all from different semantic categories and the first phoneme of each word was unique, as judged by a native speaker of Dutch. There were 20 picture grids for each background speech condition, resulting in 60 grids in total; 24 additional pictures (6 picture grids) were selected as practice stimuli from the same database.

##### Irrelevant background speech

For the Chinese word list condition (see Supplementary Material, Table A2), 120 additional Dutch nouns were selected from the MultiPic database (Duñabeitia et al., 2018) and translated into Chinese by a native Mandarin Chinese speaker. These 120 Chinese nouns were divided into 20 word lists of 6 nouns and paired with the 20 picture grids. All 20 lists were matched on the number of phonemes and number of syllables. The number of syllables was also matched between the Chinese nouns and the sets of to-be-named pictures, *t*_(305.91)_ = −1.58, *p* > .05. To avoid phonological overlap between picture naming and background speech, we designed the word lists so that the six Chinese nouns per list did not share the first phoneme, and any five consecutive Chinese nouns per list also did not share the first phoneme with the to-be-named pictures in the same ordinal position. To create practice stimuli, 12 additional Dutch nouns were selected from the same database (Duñabeitia et al., 2018) and translated into Chinese, resulting in two lists. All of the word lists were recorded by a female native Mandarin Chinese speaker in neutral prosody using Audacity software (https://www.audacityteam.org/download/) at a sample rate of 44,100 Hz. Each word list was processed using Adobe Audition (https://www.adobe.com/products/audition.html) and Praat to delete initial and final silences and compress by up to 0.74%, so that each word list lasted 8 s and there were similar periods of silence (about 700 ms) between consecutive nouns. Naming latencies for pictures can be around 1 s (e.g., Shao et al., 2014; Vitkovitch & Tyrrell, 1995), the duration (the difference from speech onset and offset of a word) of a spoken one- or two-syllable word may be up to 500 ms (e.g., Damian, 2003), and both utterance onset and articulation may be slowed in the presence of background speech. Therefore, we estimated that it takes approximately 2 s to name one picture (also see He, Meyer and Brehm 2021)), totaling 8 s per word list.

For the Chinese sentence condition (see Supplementary Material, Table A3), the 20 Chinese word lists were transformed into 20 Chinese sentences by reversing the order of nouns in the list and adding conjunctions (e.g., 和/并且, “*and*”) and prepositional phrases (e.g., 在左边/在右边; “*to the left/right of*”) to link the nouns. Again, no five consecutive Chinese nouns per sentence were phonologically related to any to-be-named pictures in the same ordinal position. The two Chinese word lists were also transformed into two Chinese sentences as practice stimuli. The same speaker recorded these in neutral prosody and they were edited in the same fashion as each Chinese word list (by stretching up to a maximum of 9.59%) to last 8 s.

To test the participants’ concentration level and compliance to wearing headphones throughout the experiment, 19 additional two-syllable Dutch nouns (4 for the practice stage, 15 for the test stage) were selected from Duñabeitia et al. (2018) to be used as attention check stimuli to be repeated back during the experiment. These were recorded by a native Dutch speaker in neutral prosody and matched on intensity, total RMS (root mean square) = −33.98 dB, in Adobe Audition.

#### Design

The type of unintelligible background speech (Chinese word list, Chinese sentences, quiet) and the difficulty of lexical selection in speech production (Name agreement: high, low) were treated as within-participant variables; both were randomised within experimental blocks and counterbalanced across participants. Items were repeated three times resulting in three blocks containing 60 trials each with one repetition of each background speech condition and picture grid. Across blocks, the same set of four pictures was paired with all three background speech conditions, and the pictures were presented in a different arrangement within each repetition. A unique order of stimulus presentation was created for each participant with the Mix programme (van Casteren & Davis, 2006), with the constraints that word lists and sentences sharing the same nouns were presented at least every three trials, and attention check trials were presented at least every five trials.

#### Procedure

Participants were tested online^1^ and received instructions that they should perform this experiment in a quiet room with the door shut and with potentially distracting electronic equipment turned off. They were asked to imagine that they were in a laboratory during the experiment, to wear headphones properly, and to set the volume of their laptops to a level that they usually use (e.g., to watch a video) and not change it during the experiment. We asked them to report their volume values before the test began.

During the experiment, a practice session of 10 trials (six test trials and four attention check trials) was followed by three blocks of experimental trials, each containing 60 test trials and five attention check trials. Participants were allowed to take a short break after each block. After completing the main portion of the experiment, participants were asked to type the value of their volume again, which allowed us to check whether they changed it during the experiment. They also were asked to fill out a questionnaire asking about their Chinese experience (see Supplementary Material, Table A4). The experiment lasted about 30 min.

**Figure 1.**
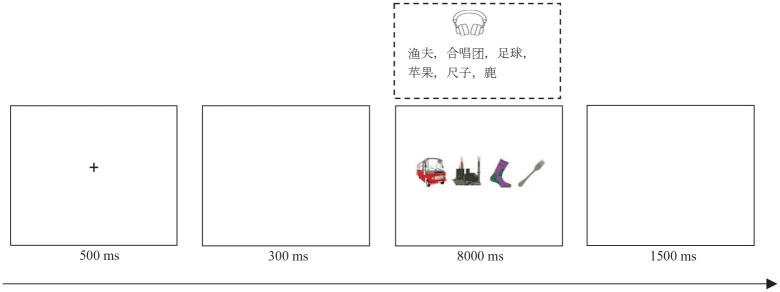
An example trial in which participants named pictures with high name agreement while ignoring a Chinese word list (translation: fisherman, choir, football, apple, ruler, deer).

Practice and experimental trials began with a fixation cross presented for 500 ms, followed by a blank screen for 300 ms. Then, a 1 × 4 grid appeared on the screen in which four pictures were presented simultaneously while a sound file played for up to 8 s. Participants named the four pictures one by one from left to right as quickly and accurately as possible while ignoring the background speech. Once finished, they clicked the mouse to end the trial, at which point a blank screen was presented for 1,500 ms. An example of a test trial is shown in Figure 1. Attention check trials were also included to test the concentration level of participants. The attention test trials shared the same structure as the test trials, but the stimulus screen was blank and an audio file of a single Dutch word was played. In these trials, participants were asked to repeat the Dutch word as quickly and accurately as possible.

#### Analyses

Seven dependent variables were coded to index naming performance. This provides a full description of the many ways production performance can be disrupted. Production *accuracy* reflects the proportion of trials where all four pictures were named correctly. Picture names were coded as correct if they matched any of the multiple names given to the picture in the MultiPic database (Duñabeitia et al., 2018); if they were diminutive versions of one of those names (e.g., *munt* “coin” named as *muntje* “little coin”), or if they were judged reasonable by trained research assistants (e.g., *kruk* “stool” named as *stoel* “chair”).

For trials on which all pictures were named correctly and which had no hesitations or self-corrections (hereafter, “fully correct trials”), we calculated four time-based measures. *Onset latency* was defined as the interval from the onset of stimulus presentation to onset of the utterance, and indexes the beginning stages of speech planning. *Utterance duration* was defined as the interval between the onset of the first picture name and the offset of the fourth picture name, and reflects how long participants took to produce all four picture names. *Total pause time* was defined as the sum of all pauses between object names, and indexes the planning done between producing responses. *Articulation time* was defined as the sum of the articulation durations of all four picture names, and reflects processing during articulations.

For fully correct trials, we also examined how participants grouped their four responses. Since earlier studies of spontaneous speech coded silent durations longer than 200 ms as silent pauses (e.g., Heldner & Edlund, 2010), we coded responses with 200 ms or less between them as a single response chunk. Two measures were derived: *Total chunk number* refers to how many response chunks participants made on one trial, with a larger number meaning more separate planning units for production. *First chunk length* refers to how many names participants produced in their initial response, and provides a measure of how much information participants planned before starting to speak.

To quantify the magnitude of all effects, Bayesian mixed-effect models (Nicenboim & Vasishth, 2016) were conducted in R version 4.0.3 (R Core Team, 2020) with the package *brms* (version 2.14.4, Bürkner, 2017). Predictors were name agreement (high/low) and the type of background speech (Chinese word list/Chinese sentence/quiet). Name agreement (high/low) was contrast coded with (0.5, −0.5). Two contrasts were made for the type of background speech: the first was coded with (0.25, 0.25, −0.5) to compare the two Chinese speech conditions (word list and sentence) with the quiet condition, and the second was coded with (0.5, −0.5, 0) to compare the Chinese word list and Chinese sentence conditions. The random effect structure for the models included random intercepts for participants and items, and random slopes for name agreement and the type of background speech by participants and items. Separate models were fitted for each dependent measure. All models had four chains and each chain had 24,000 iterations depending on model convergence (listed in model output tables). We used a warm-up (or burn-in) period of 2,000 iterations in each chain, which means we removed the data based on the first 2,000 iterations in order to correct the initial sampling bias.

All models used weak, widely spread priors that would be consistent with a range of null to moderate effects. The model of accuracy used family *Bernoulli* combined with a *logit* link, with a Student-*t* prior with 1 degree of freedom and a scale parameter of 2.5. The models of log-transformed onset latency, log-transformed utterance duration, and log-transformed articulation time used a weak normal prior with an *SD* of 0.2, and the model of log-transformed total pause time used a weak normal prior with an *SD* of 1. These models were performed using the family *Gaussian* and *identity* link. Total chunk number and first chunk length had weak normal priors centred at zero with an *SD* of 1, and used family *Poisson* combined with the *log* link. All models were run until the R-hat value for each parameter was 1.00, indicating convergence.

For these models, the size of reported betas reflects estimated effect sizes, with larger absolute values of betas reflecting larger effects. We reported the parameters for which 95% credible intervals (hereafter, Cr.I) do not contain zero, which is analogous to the frequentist null hypothesis significance test: the parameter has a non-zero effect with high certainty. We also reported any parameters for which the point estimate for the beta is about twice the size of its error, as this suggests that the estimated effect is large compared with the uncertainty around it. We also reported the posterior probability of all weak effects, indicating the proportion of samples with a value equal to or above the beta estimate.

### Results

Six participants were removed from further analyses: three did not run the experiments successfully due to a bad internet connection, two gave no responses on attention check trials, and one had too much Chinese experience as indicated by their responses on the Chinese experience questionnaire. The data from the remaining 44 participants were checked for errors, removing from analysis any trials with implausible names (e.g., *koekje* “cookie” named as *virus*), hesitations (e.g., *komkommer* “cucumber” named as *kom . . . komkommer*), self-corrections (e.g., *komkommer* “cucumber” misnamed as *courgette . . . komkommer* “courgette . . . cucumber”), and any trials where objects were omitted or named in the wrong order. The exclusion of these inaccurate trials resulted in a loss of 13.7% of the data (range by participants: 1.1%–30% of removed trials). Then, any onset latencies below 200 ms were removed from this analysis, resulting in a loss of 0.47% of the data. Any total pause times below 20 ms were also removed from this analysis, resulting in a loss of 12.98% of the data. Finally, any data points more than 2.5 *SD*s below or above the mean values were removed for each time measure (1.87% for log-transformed onset latency, 0.86% for log-transformed utterance duration, 0.97% for log-transformed total pause time, and 1.33% for log-transformed articulation time). Descriptive statistics appear in Table 2.

**Table 2.** Means and standard deviations of the dependent variables by name agreement and the type of background speech in Experiment 1.

	High NA			Low NA		
	Chinese Word List	Chinese sentence	Quiet	Chinese word list	Chinese sentence	Quiet
Accuracy	91%	91%	92%	82%	82%	81%
Onset latency (ms)	1,246(462)	1,279 (522)	1,198 (408)	1,434 (579)	1,413 (539)	1,345 (486)
Utterance duration (ms)	2,868(790)	2,868 (771)	2,791(765)	3,475 (1,062)	3,482(1,025)	3,392 (970)
Total pause time (ms)	685(621)	662 (590)	645 (582)	1,078 (860)	1,043 (790)	1,040 (805)
Articulation time (ms)	2,309(431)	2,332 (429)	2,246 (392)	2,518 (498)	2,536 (522)	2,450 (476)
Total chunk number	1.9 (1.0)	1.9 (1.0)	1.9 (1.0)	2.3 (1.1)	2.4 (1.1)	2.4 (1.1)
First chunk length	2.7 (1.3)	2.7 (1.3)	2.8 (1.3)	2.3 (1.3)	2.2 (1.2)	2.2 (1.2)

*Note.* Standard deviations are given in parentheses. All time and chunking measures reflect fully correct trials only. NA: name agreement.

#### Attention check

The mean accuracy for attention check responses was 97% (range by participants: 73%–100%), showing that participants’ attention levels were good and that they indeed heard the background speech.

#### Accuracy

Participants produced sensible responses on 86% of the naming trials. As shown in Table 3, a Bayesian mixed-effect model showed that accuracy was considerably lower for low name agreement pictures than high name agreement pictures (β = .099, *SE* = .025, 95% Cr.I = [0.051, 0.147]), but it was not influenced by the type of background speech. Name agreement and the type of background speech did not interact.

**Table 3.** Results of Bayesian mixed-effect models for all dependent variables in Experiment 1.

	Estimate	Est. error	95% Cr. I		Effective samples
			Lower	Upper	
Accuracy					
Population-level effects					
Intercept	0.863	0.017	0.83	0.895	32,170
Name agreement	**0.099**	**0.025**	**0.051**	**0.147**	**59,697**
Speech vs. quiet	0	0.014	–0.028	0.029	107,958
Word List vs. Sentence	0.003	0.011	–0.019	0.025	131,954
NA × (S vs. Q)	–0.02	0.028	–0.076	0.036	107,878
NA × (WL vs. S)	0.001	0.022	–0.042	0.045	134,552
Group-level effects					
Participants					
sd(Intercept)	0.075	0.009	0.06	0.095	27,257
sd(NA)	0.043	0.01	0.024	0.064	54,647
sd(S vs. Q)	0.016	0.012	0.001	0.043	48,050
sd(WL vs. S)	0.012	0.009	0.001	0.033	56,746
sd(NA × (S vs. Q))	0.021	0.016	0.001	0.061	69,866
sd(NA × (WL vs. S))	0.023	0.017	0.001	0.065	55,462
Items					
sd(Intercept)	0.058	0.02	0.016	0.092	6,156
sd(NA)	0.117	0.04	0.033	0.184	6,086
sd(S vs. Q)	0.05	0.018	0.011	0.085	20,580
sd(WL vs. S)	0.03	0.018	0.002	0.066	16,829
sd(NA × (S vs. Q))	0.099	0.037	0.023	0.17	22,166
sd(NA × (WL vs. S))	0.06	0.036	0.003	0.133	17,133
Log-transformed onset latency					
Population-level effects					
Intercept	7.133	0.028	7.078	7.188	5,293
Name agreement	**–0.122**	**0.014**	**–0.149**	**–0.095**	**48,510**
Speech vs. quiet	*0.064*	*0.038*	*–0.011*	*0.138*	*49,911*
Word list vs. sentence	–0.002	0.037	–0.074	0.071	47,960
NA × (S vs. Q)	–0.006	0.07	–0.144	0.132	50,854
NA × (WL vs. S)	–0.014	0.069	–0.15	0.122	56,068
Group-level effects					
Participants					
sd(Intercept)	0.177	0.02	0.143	0.223	10,270
sd(NA)	0.029	0.011	0.005	0.051	18,616
sd(S vs. Q)	0.077	0.015	0.049	0.109	31,488
sd(WL vs. S)	0.05	0.013	0.024	0.077	24,869
sd(NA × (S vs. Q))	0.035	0.025	0.001	0.091	27,704
sd(NA × (WL vs. S))	0.048	0.027	0.003	0.105	21,254
Items					
sd(Intercept)	0.029	0.012	0.004	0.049	2,331
sd(NA)	0.058	0.024	0.008	0.098	2,319
sd(S vs. Q)	0.173	0.095	0.008	0.311	1,284
sd(WL vs. S)	0.177	0.1	0.006	0.316	1,181
sd(NA × (S vs. Q))	0.345	0.189	0.016	0.622	1,222
sd(NA × (WL vs. S))	0.325	0.202	0.011	0.626	1,228
Log-transformed utterance duration					
Population-level effects					
Intercept	8.021	0.023	7.974	8.066	6,414
Name agreement	**–0.191**	**0.02**	**–0.231**	**–0.151**	**39,748**
Speech vs. quiet	0.029	0.026	–0.022	0.08	54,056
Word list vs. sentence	–0.003	0.022	–0.046	0.04	51,599
NA × (S vs. Q)	0.018	0.05	–0.081	0.117	56,494
NA × (WL vs. S)	0.005	0.044	–0.081	0.091	49,868
Group-level effects					
Participants					
sd(Intercept)	0.142	0.016	0.115	0.178	12,242
sd(NA)	0.064	0.009	0.047	0.084	35,908
sd(S vs. Q)	0.014	0.01	0.001	0.036	35,029
sd(WL vs. S)	0.01	0.007	0	0.026	45,776
sd(NA × (S vs. Q))	0.019	0.014	0.001	0.054	49,185
sd(NA × (WL vs. S))	0.04	0.02	0.004	0.081	31,111
Items					
sd(Intercept)	0.04	0.023	0.002	0.074	1,565
sd(NA)	0.081	0.045	0.004	0.148	1,643
sd(S vs. Q)	0.125	0.055	0.015	0.21	3,193
sd(WL vs. S)	0.111	0.036	0.037	0.173	5,059
sd(NA × (S vs. Q))	0.251	0.109	0.032	0.422	3,182
sd(NA × (WL vs. S))	0.222	0.073	0.072	0.346	4,698
Log-transformed total pause time					
Population-level effects					
Intercept	6.274	0.081	6.115	6.432	7,041
Name agreement	**–0.574**	**0.058**	**–0.687**	**–0.46**	**43,884**
Speech vs. quiet	0.009	0.07	–0.127	0.147	67,063
Word list vs. sentence	0.017	0.064	–0.108	0.143	58,586
NA × (S vs. Q)	0.039	0.134	–0.224	0.304	69,382
NA × (WL vs. S)	0.033	0.126	–0.216	0.283	62,853
Group-level effects					
Participants					
sd(Intercept)	0.508	0.058	0.41	0.635	13,162
sd(NA)	0.177	0.033	0.116	0.247	43,499
sd(S vs. Q)	0.122	0.052	0.017	0.222	26,954
sd(WL vs. S)	0.067	0.04	0.004	0.152	31,799
sd(NA × (S vs. Q))	0.078	0.06	0.003	0.223	53,517
sd(NA × (WL vs. S))	0.126	0.08	0.006	0.298	32,126
Items					
sd(Intercept)	0.107	0.063	0.004	0.204	2,282
sd(NA)	0.222	0.124	0.01	0.409	2,251
sd(S vs. Q)	0.293	0.14	0.023	0.518	3,763
sd(WL vs. S)	0.292	0.102	0.078	0.469	6,780
sd(NA × (S vs. Q))	0.59	0.279	0.049	1.038	3,738
sd(NA × (WL vs. S))	0.579	0.205	0.151	0.935	6,811
Log-transformed articulation time					
Population-level effects					
Intercept	7.768	0.019	7.731	7.805	5,872
Name agreement	**–0.085**	**0.02**	**–0.125**	**–0.046**	**46,351**
Speech vs. quiet	**0.038**	**0.014**	**0.01**	**0.066**	**61,569**
Word list vs. sentence	–0.007	0.012	–0.031	0.017	64,224
NA × (S vs. Q)	0.007	0.027	–0.046	0.06	66,049
NA × (WL vs. S)	–0.003	0.024	–0.05	0.044	62,948
Group-level effects					
Participants					
sd(Intercept)	0.108	0.013	0.087	0.136	11,302
sd(NA)	0.053	0.007	0.041	0.069	28,988
sd(S vs. Q)	0.029	0.008	0.011	0.045	20,619
sd(WL vs. S)	0.008	0.005	0	0.02	35,991
sd(NA × (S vs. Q))	0.014	0.011	0.001	0.039	41,441
sd(NA × (WL vs. S))	0.021	0.014	0.001	0.051	21,175
Items					
sd(Intercept)	0.042	0.026	0.001	0.078	1,378
sd(NA)	0.083	0.051	0.003	0.157	1,380
sd(S vs. Q)	0.06	0.036	0.002	0.113	1,763
sd(WL vs. S)	0.055	0.029	0.003	0.098	1,923
sd(NA × (S vs. Q))	0.121	0.071	0.005	0.225	1,729
sd(NA × (WL vs. S))	0.106	0.059	0.005	0.195	1,932
Total chunk number					
Population-level effects					
Intercept	0.715	0.041	0.635	0.795	9,365
Name agreement	**–0.252**	**0.025**	**–0.301**	**–0.203**	**52,559**
Speech vs. quiet	–0.016	0.035	–0.085	0.053	74,601
Word list vs. sentence	–0.017	0.029	–0.074	0.040	79,456
NA × (S vs. Q)	0.014	0.070	–0.123	0.152	77,761
NA × (WL vs. S)	0.009	0.058	–0.105	0.123	78,972
Group-level effects					
Participants					
sd(Intercept)	0.256	0.030	0.206	0.321	15,391
sd(NA)	0.062	0.021	0.020	0.104	46,312
sd(S vs. Q)	0.023	0.018	0.001	0.067	62,627
sd(WL vs. S)	0.020	0.016	0.001	0.058	63,929
sd(NA × (S vs. Q))	0.049	0.037	0.002	0.139	64,075
sd(NA × (WL vs. S))	0.043	0.033	0.002	0.122	61,696
Items					
sd(Intercept)	0.035	0.020	0.002	0.073	8,804
sd(NA)	0.070	0.040	0.004	0.146	7,966
sd(S vs. Q)	0.124	0.058	0.012	0.229	9,285
sd(WL vs. S)	0.102	0.043	0.014	0.183	13,656
sd(NA × (S vs. Q))	0.246	0.116	0.020	0.458	9,163
sd(NA × (WL vs. S))	0.202	0.087	0.025	0.365	13,743
First chunk length					
Population-level effects					
Intercept	0.863	0.042	0.781	0.946	11,967
Name agreement	**0.218**	**0.025**	**0.168**	**0.268**	**96,798**
Speech vs. quiet	–0.012	0.034	–0.077	0.055	95,932
Word list vs. sentence	0.013	0.030	–0.046	0.072	92,168
NA × (S vs. Q)	–0.030	0.067	–0.162	0.101	95,948
NA × (WL vs. S)	–0.027	0.060	–0.145	0.091	95,897
Group-level effects					
Participants					
sd(Intercept)	0.262	0.031	0.210	0.330	19,220
sd(NA)	0.022	0.016	0.001	0.061	50,297
sd(S vs. Q)	0.025	0.019	0.001	0.069	64,357
sd(WL vs. S)	0.023	0.018	0.001	0.065	61,516
sd(NA × (S vs. Q))	0.047	0.036	0.002	0.135	64,675
sd(NA × (WL vs. S))	0.043	0.033	0.002	0.122	63,963
Items					
sd(Intercept)	0.047	0.025	0.003	0.090	5,967
sd(NA)	0.094	0.050	0.005	0.179	5,836
sd(S vs. Q)	0.124	0.053	0.015	0.221	11,407
sd(WL vs. S)	0.116	0.042	0.028	0.195	19,228
sd(NA × (S vs. Q))	0.249	0.106	0.031	0.442	13,355
sd(NA × (WL vs. S))	0.230	0.085	0.051	0.389	18,080

NA: name agreement; WL: word list; S: sentence; Q: quiet.

Models for all dependent variables were run for 24,000 iterations. Bolded values indicate effects where the 95% Cr.I does not contain zero.

#### Onset latency

**Figure 2.**
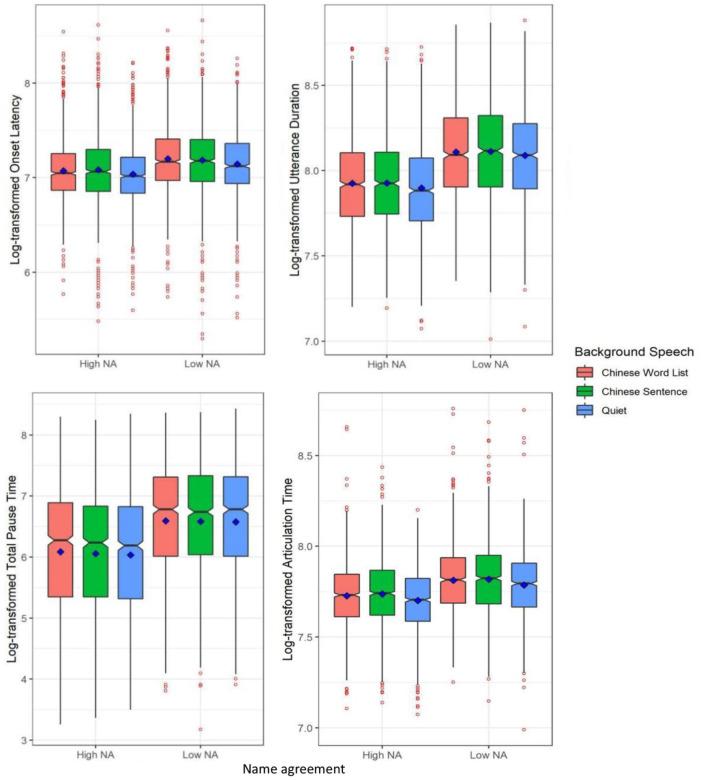
Log-transformed onset latency (top-left), log-transformed utterance duration (top-right), log-transformed total pause time (bottom-left), and log-transformed articulation time (bottom-right) split by name agreement (NA: high, low) and the type of background speech (Chinese word list, Chinese sentence, Quiet) in Experiment 1. Blue squares represent condition means and red points reflect outliers.

As shown in Table 3 and the left panel of Figure 2, a Bayesian mixed-effect model showed that log-transformed onset latency was affected by name agreement: it took participants longer to plan names for low name agreement pictures than high name agreement pictures (β = −.122, *SE* = 0.014, 95% Cr.I = [−0.149, −0.095]). There was moderate evidence for the first contrast (Chinese vs. Quiet) of background speech, showing that the log-transformed onset latencies in the two Chinese speech conditions (word list and sentence) were slower than in the quiet condition (β = .064, *SE* = 0.038, 95% Cr.I = [−0.011, 0.138]). Note that while the 95% Cr.I contains zero, the point estimate is high relative to the error around it, and 96% of the posterior distribution around the estimated effect is above zero. Name agreement and the type of background speech did not interact.

#### Utterance duration

As shown in Table 3 and the right panel of Figure 2, a Bayesian mixed-effect model showed that the log-transformed utterance duration was longer for low name agreement pictures than high name agreement pictures (β = −.191, *SE* = 0.02, 95% Cr.I = [−0.231, −0.151]), but it was not influenced by the type of background speech. Again, name agreement and the type of background speech did not interact.

#### Total pause time

As shown in Table 3 and the left panel of Figure 2, a Bayesian mixed-effect model showed that the results for this measurement patterned in the same way as the log-transformed utterance duration. The log-transformed total pause time was considerably longer for low name agreement pictures than high name agreement pictures (β = −0.574, *SE* = 0.058, 95% Cr.I = [−0.687, −0.46]), but it did not vary with the type of background speech. Name agreement and the type of background speech did not interact.

#### Articulation time

As shown in Table 3 and the right panel of Figure 2, a Bayesian mixed-effect model showed that log-transformed articulation time was influenced by both name agreement and the type of background speech: It was significantly longer for low name agreement pictures than high name agreement pictures (β = −.085, *SE* = 0.02, 95% Cr.I = [−0.125, −0.046]), and it was reliably longer in the two Chinese speech conditions (word list and sentence) than in the quiet condition (β = 0.038, *SE* = 0.014, 95% Cr.I = [0.01, 0.066]). Again, name agreement did not interact with the type of background speech.

#### Total chunk number

**Figure 3.**
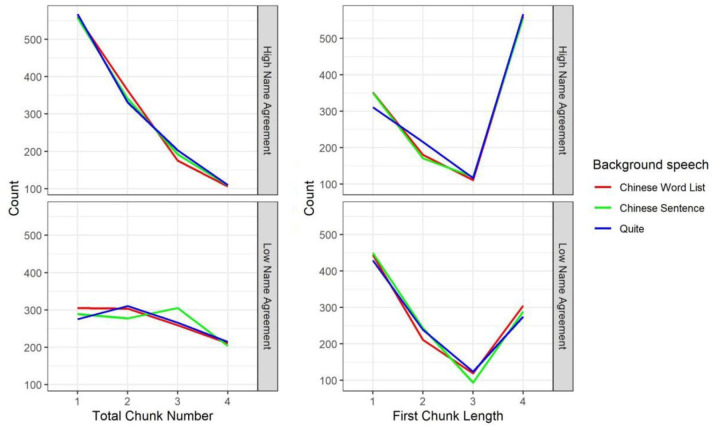
Total chunk number (left) and first chunk length (right) split by name agreement (NA: high, low) and the type of background speech (Chinese word list, Chinese sentence, Quiet) in Experiment 1.

As shown in Table 3 and the left panel of Figure 3, a Bayesian mixed-effect model showed that participants grouped their responses in more chunks for low name agreement pictures than high name agreement pictures (β = −.252, *SE* = −0.025, 95% Cr.I = [−0.301, −0.203]). There was no interaction between name agreement and the type of background speech.

#### First chunk length

As shown in Table 3 and the right panel of Figure 3, a Bayesian mixed-effect model showed that participants planned fewer names in their first response chunk for low name agreement pictures than high name agreement pictures (β = .218, *SE* = 0.025, 95% Cr.I = [0.168, 0.258]). First chunk length was not affected by the type of background speech and there was no interaction between name agreement and the type of background speech.

### Interim discussion

This experiment provides support for phonological disruption and specific attention capture impacting speech production. Consistent with the phonological disruption view (Salamé & Baddeley, 1982, 1989), the presence of Chinese background speech (word lists and sentences) increased articulation time significantly, but only had a weak impact on speech onset latencies relative to a quiet condition. Consistent with the specific attention capture view (Eimer et al., 1996), there was no difference between the Chinese word list and Chinese sentence conditions on any dependent measures. Finally, name agreement had a main effect on all dependent measures (as in Alario et al., 2004; He et al., 2021; Shao et al., 2014), but did not interact with the type of Chinese background speech, consistent with the automatic stimulus-aspecific disruption proposal by Hughes (2014).

## Experiment 2

Experiment 1 demonstrated clear phonological disruption and specific attention capture effects on unintelligible background speech. However, it is unclear whether these patterns generalise to intelligible background speech. Thus, we extended our investigation to an intelligible-background-speech context by replacing Chinese speech with Dutch speech in Experiment 2. Here, both the phonological and semantic disruption views (Martin et al., 1988; Salamé & Baddeley, 1982, 1989) predict that Dutch speech (word lists and sentences) should disrupt speech production relative to a quiet condition. The aspecific attention capture view (Eimer et al., 1996) predicts there may be more interference in the Dutch word list condition (because of pauses it contains), while the specific attention capture view (Eimer et al., 1996) predicts there may be more disruption in the Dutch sentence condition (due to richer representation recruitment); combined, we make relatively weak predictions under the attention capture variants. Finally, following the claim that the stimulus-specific auditory distraction should be reduced or eliminated by an increase in attention engagement because it requires central attention and cognitive control (Hughes, 2014; Marsh et al., 2018), we predicted that planning low name agreement pictures would reduce the processing—and thus interference—of Dutch background speech.

### Method

#### Participants

We recruited 47 native Dutch speakers (33 females, *M*_age_ = 26 years, range: 18–39 years) from the same participant pool as Experiment 1. This sample size was selected because power simulations (see https://osf.io/wuafh/ for scripts) showed that 46 participants and 144 items (an 80% accuracy rate) would provide 96% power to measure an interaction between the type of background speech and name agreement on the measurement of utterance duration of 20 ms or smaller (*SD* = 900 ms) for low name agreement pictures and 60 ms or larger (*SD* = 900 ms) for high name agreement pictures. All participants reported normal or corrected-to-normal vision and no speech or hearing problems. They signed an online informed consent form and received a payment of €6 for their participation. The study was approved by the ethics board of the Faculty of Social Sciences of Radboud University.

#### Apparatus

The same apparatus was used as in Experiment 1.

#### Materials

##### Visual stimuli

As in Experiment 1.

##### Irrelevant background speech

For the Dutch word lists (see Supplementary Material, Table B1), the 120 nouns from Experiment 1 were used in Dutch, and matched with picture names on WF, number of syllables, number of phonemes, age-of-acquisition, and word prevalence. To pair with the set of 20 picture grids, these 120 Dutch nouns were divided into 20 word lists of 6 nouns, each list matched on WF and number of syllables. To equate the amount of semantic and phonological overlap across trials between speech planning and auditory background speech, we made sure that six Dutch nouns per word list were neither semantically nor phonologically related to each other, as described in Experiment 1. In addition, 12 Dutch versions of nouns from Experiment 1 were used as practice stimuli, resulting in two Dutch word lists. All of the Dutch word lists were recorded by a female native Dutch speaker^2^ in neutral prosody and further edited as the Chinese word lists were to last 8 s each with similar silent periods (about 700 ms) between consecutive nouns, by stretching by up to 9.38%.

For the Dutch sentence condition (see Supplementary Material, Table B2), the 20 Dutch word lists were transformed into 20 Dutch sentences as in Experiment 1 by reversing the order of the nouns and then combining them with conjunctions (e.g., *en* “and”) and prepositional phrases (e.g., *bevinden zich links/rechts van* “are to the left/right of”). The two Dutch word lists were also translated into two Dutch sentences as practice stimuli. The same female native Dutch speaker recorded these sentences in neutral prosody. Sentences were edited to last 8 s each by stretching by up to 14.29%. The same 19 attention catch trials (15 as test stimuli, 4 as practice stimuli) from Experiment 1 were also included. All auditory files were matched on intensity (total RMS = −33.98 dB) in Adobe Audition.

#### Design

The design was identical to Experiment 1.

#### Procedure

The procedure was identical to Experiment 1 except that participants did not fill out the questionnaire of Chinese experience.^3^

#### Analyses

The analysis was the same as Experiment 1.

### Results

Six participants were removed from further analyses: one had no audio recordings, three had no responses for attention check trials, one had also participated in Experiment 1, and one had extremely poor-quality audio recordings. The data from the remaining 41 participants were checked for errors as described in Experiment 1. The exclusion of these inaccurate trials resulted in a loss of 12.7% of data (range by participants: 2.8%–42% of removed trials). Then, any data points below 200 ms were removed for onset latency, resulting in a loss of 0.02% of the data. Any data points below 20 ms were also removed for the total pause time measure, resulting in a loss of 12.17% of the data. Finally, any data points more than 2.5 *SDs* below or above the mean values were removed for the time measures (1.61% for log-transformed onset latency, 0.85% for log-transformed utterance duration, 1.01% for log-transformed total pause time, and 1.18% for log-transformed articulation time). Descriptive statistics of all dependent variables are shown in Table 4.

**Table 4.** Means and standard deviations of the dependent variables by name agreement and the type of background speech in Experiment 2.

	High NA			Low NA		
	Dutch word list	Dutch sentence	Quiet	Dutch word list	Dutch sentence	Quiet
Accuracy	92%	92%	93%	82%	82%	84%
Onset latency (ms)	1,304 (496)	1,300 (493)	1,195 (362)	1,451 (568)	1,486 (611)	1,392 (492)
Utterance duration (ms)	2,864 (859)	2,871 (872)	2,690 (776)	3,481 (1,028)	3,463 (1,078)	3,474 (1,087)
Total pause time (ms)	771 (759)	726 (745)	632 (636)	1,090 (877)	1,072 (903)	1,160 (909)
Articulation time (ms)	2,260 (393)	2,274 (415)	2,172 (387)	2,484 (467)	2,482 (482)	2,392 (458)
Total chunk number	1.9 (1.0)	1.9 (1.0)	1.9 (1.0)	2.4 (1.0)	2.4 (1.1)	2.5 (1.1)
First chunk length	2.7 (1.3)	2.8 (1.3)	2.8 (1.3)	2.2 (1.2)	2.3 (1.2)	2.2 (1.2)

NA: name agreement. Standard deviations are given in parentheses. All time and chunking measures reflect fully correct trials only.

#### Attention check

The mean accuracy for attention check responses was 98% (range by participants: 73% - 100%), showing that participants indeed processed the background speech during the experiment.

#### Accuracy

Participants produced the intended responses on 87% of the naming trials. As shown in Table 5, a Bayesian mixed-effect model showed that accuracy was lower for low name agreement pictures than high name agreement pictures (β = 1.061, *SE* = 0.223, 95% Cr.I = [0.630, 1.506]), but it was not affected by the type of background speech. Name agreement and the type of background speech did not interact.

**Table 5.** Results of Bayesian mixed-effect models for all dependent variables in Experiment 2.

	Estimate	Est.error	95% Cr. I		Effective samples
			Lower	Upper	
Accuracy					
Population-level effects					
Intercept	2.295	0.165	1.974	2.628	29,013
Name agreement	**1.061**	**0.223**	**0.630**	**1.506**	**79,513**
Speech vs. quiet	–0.043	0.142	–0.328	0.230	118,039
Word list vs. sentence	0.016	0.123	–0.231	0.256	109,284
NA × (S vs. Q)	–0.134	0.275	–0.669	0.412	118,838
NA × (WL vs. S)	0.063	0.246	–0.416	0.553	112,914
Group-level effects					
Participants					
sd(Intercept)	0.812	0.103	0.634	1.038	28,016
sd(NA)	0.317	0.135	0.043	0.582	25,107
sd(S vs. Q)	0.171	0.123	0.007	0.455	45,424
sd(WL vs. S)	0.125	0.093	0.005	0.345	54,483
sd(NA × (S vs. Q))	0.220	0.169	0.008	0.630	64,394
sd(NA × (WL vs. S))	0.236	0.178	0.009	0.663	53,301
Items					
sd(Intercept)	0.478	0.265	0.020	0.868	2,980
sd(NA)	0.901	0.531	0.034	1.714	3,066
sd(S vs. Q)	0.340	0.189	0.021	0.715	19,407
sd(WL vs. S)	0.315	0.187	0.017	0.692	18,572
sd(NA × [S vs. Q])	0.652	0.371	0.039	1.394	21,918
sd(NA × (WL vs. S))	0.601	0.366	0.030	1.338	18,389
Log-transformed onset latency					
Population-level effects					
Intercept	7.161	0.028	7.105	7.216	5,610
Name agreement	**–0.128**	**0.014**	**–0.155**	**–0.1**	**60,813**
Speech vs. quiet	*0.076*	*0.04*	*–0.003*	*0.155*	*61,479*
Word list vs. sentence	–0.004	0.046	–0.096	0.086	65,617
NA × (S vs. Q)	0.04	0.074	–0.104	0.187	64,085
NA × (WL vs. S)	0.022	0.086	–0.147	0.19	66,181
Group-level effects					
Participants					
sd(Intercept)	0.171	0.02	0.136	0.217	12,128
sd(NA)	0.024	0.011	0.003	0.044	22,175
sd(S vs. Q)	0.05	0.014	0.021	0.078	26,754
sd(WL vs. S)	0.028	0.014	0.002	0.054	20,076
sd(NA × (S vs. Q))	0.027	0.02	0.001	0.074	39,897
sd(NA × (WL vs. S))	0.026	0.018	0.001	0.067	39,453
Items					
sd(Intercept)	0.029	0.016	0.001	0.053	1,183
sd(NA)	0.059	0.031	0.003	0.107	1,196
sd(S vs. Q)	0.184	0.106	0.008	0.339	1,012
sd(WL vs. S)	0.233	0.117	0.016	0.405	2,193
sd(NA × (S vs. Q))	0.376	0.213	0.015	0.68	1,029
sd(NA × (WL vs. S))	0.454	0.237	0.029	0.807	2,111
Log-transformed utterance duration					
Population-level effects					
Intercept	8.012	0.028	7.957	8.067	4,298
Name agreement	**–0.215**	**0.022**	**–0.257**	**–0.172**	**34,356**
Speech vs. quiet	*0.050*	*0.031*	*–0.012*	*0.111*	*48,720*
Word list vs. sentence	0.005	0.024	–0.042	0.052	54,738
NA × (S vs. Q)	0.070	0.060	–0.047	0.187	50,417
NA × (WL vs. S)	–0.007	0.047	–0.100	0.085	58,527
Group-level effects					
Participants					
sd(Intercept)	0.171	0.021	0.136	0.216	11,188
sd(NA)	0.073	0.011	0.054	0.097	31,638
sd(S vs. Q)	0.045	0.014	0.014	0.072	16,224
sd(WL vs. S)	0.008	0.006	0.000	0.023	55,147
sd(NA × (S vs. Q))	0.039	0.027	0.002	0.097	21,573
sd(NA × (WL vs. S))	0.019	0.014	0.001	0.054	45,545
Items					
sd(Intercept)	0.044	0.023	0.002	0.078	1,561
sd(NA)	0.085	0.046	0.004	0.155	1,554
sd(S vs. Q)	0.151	0.065	0.021	0.253	2,658
sd(WL vs. S)	0.112	0.059	0.006	0.200	1,808
sd(NA × (S vs. Q))	0.301	0.130	0.040	0.504	2,617
sd(NA × (WL vs. S))	0.225	0.119	0.012	0.401	1,766
Log-transformed total pause time					
Population-level effects					
Intercept	6.298	0.09	6.12	6.476	8,463
Name agreement	**–0.599**	**0.072**	**–0.741**	**–0.458**	**50,058**
Speech vs. quiet	0.055	0.086	–0.114	0.224	74,556
Word list vs. sentence	0.059	0.068	–0.075	0.194	8,7601
NA × (S vs. Q)	*0.28*	*0.173*	*–0.06*	*0.621*	*74,891*
NA × (WL vs. S)	–0.006	0.137	–0.275	0.263	88,114
Group-level effects					
Participants					
sd(Intercept)	0.542	0.065	0.432	0.687	16,813
sd(NA)	0.28	0.042	0.207	0.373	38,849
sd(S vs. Q)	0.078	0.051	0.004	0.188	27,262
sd(WL vs. S)	0.035	0.027	0.001	0.099	55,607
sd(NA × (S vs. Q))	0.28	0.12	0.035	0.51	25,088
sd(NA × (WL vs. S))	0.117	0.078	0.005	0.29	35,367
Items					
sd(Intercept)	0.125	0.067	0.007	0.227	2,808
sd(NA)	0.249	0.134	0.014	0.455	2,789
sd(S vs. Q)	0.401	0.163	0.067	0.665	4,686
sd(WL vs. S)	0.297	0.168	0.012	0.549	2,653
sd(NA × (S vs. Q))	0.786	0.326	0.123	1.322	4,524
sd(NA × (WL vs. S))	0.589	0.337	0.024	1.099	2,693
Log-transformed articulation time					
Population-level effects					
Intercept	7.744	0.021	7.704	7.785	8,367
Name agreement	**–0.093**	**0.020**	**–0.133**	**–0.054**	**63,460**
Speech vs. quiet	**0.054**	**0.016**	**0.023**	**0.085**	**97,570**
Word list vs. sentence	–0.003	0.013	–0.029	0.022	100,970
NA × (S vs. Q)	0.010	0.030	–0.048	0.069	103,634
NA × (WL vs. S)	0.000	0.026	–0.050	0.051	101,332
Group-level effects					
Participants					
sd(Intercept)	0.120	0.014	0.096	0.152	16,082
sd(NA)	0.055	0.008	0.042	0.071	33,143
sd(S vs. Q)	0.031	0.007	0.018	0.046	24,300
sd(WL vs. S)	0.007	0.005	0.000	0.018	43,960
sd(NA × (S vs. Q))	0.033	0.017	0.002	0.067	20,736
sd(NA × (WL vs. S))	0.017	0.011	0.001	0.041	37,705
Items					
sd(Intercept)	0.042	0.025	0.001	0.078	1,772
sd(NA)	0.083	0.051	0.003	0.156	1,798
sd(S vs. Q)	0.066	0.040	0.002	0.124	1,927
sd(WL vs. S)	0.058	0.035	0.002	0.108	2,217
sd(NA × (S vs. Q))	0.130	0.080	0.004	0.247	1,977
sd(NA × (WL vs. S))	0.116	0.069	0.004	0.217	2,209
Total chunk number					
Population-level effects					
Intercept	0.728	0.041	0.647	0.808	8,660
Name agreement	**–0.266**	**0.030**	**–0.325**	**–0.208**	**41,811**
Speech vs. quiet	–0.003	0.037	–0.077	0.071	73,370
Word list vs. sentence	0.015	0.030	–0.045	0.074	77,365
NA × (S vs. Q)	0.070	0.075	–0.078	0.217	74,377
NA × (WL vs. S)	0.014	0.061	–0.105	0.133	79,264
Group-level effects					
Participants					
sd(Intercept)	0.246	0.030	0.196	0.312	15,554
sd(NA)	0.086	0.022	0.045	0.132	47,199
sd(S vs. Q)	0.024	0.019	0.001	0.070	62,041
sd(WL vs. S)	0.020	0.015	0.001	0.057	68,947
sd(NA × (S vs. Q))	0.051	0.040	0.002	0.148	61,109
sd(NA × (WL vs. S))	0.040	0.031	0.002	0.114	70,155
Items					
sd(Intercept)	0.047	0.026	0.002	0.092	4,816
sd(NA)	0.094	0.052	0.005	0.184	4,829
sd(S vs. Q)	0.140	0.066	0.012	0.257	7,236
sd(WL vs. S)	0.102	0.057	0.005	0.204	6,819
sd(NA × (S vs. Q))	0.278	0.132	0.023	0.512	7,343
sd(NA × (WL vs. S))	0.201	0.114	0.010	0.407	6,661
First chunk length					
Population-level effects					
Intercept	0.858	0.045	0.767	0.948	8,363
Name agreement	**0.237**	**0.027**	**0.183**	**0.291**	**74,876**
Speech vs. quiet	–0.008	0.043	–0.092	0.076	64,681
Word list vs. sentence	–0.022	0.036	–0.093	0.048	70,214
NA × (S vs. Q)	–0.090	0.085	–0.257	0.078	65,380
NA × (WL vs. S)	–0.005	0.072	–0.146	0.137	70,142
Group-level effects					
Participants					
sd(Intercept)	0.272	0.034	0.214	0.346	17,057
sd(NA)	0.030	0.021	0.001	0.079	35,240
sd(S vs. Q)	0.026	0.019	0.001	0.073	58,663
sd(WL vs. S)	0.021	0.016	0.001	0.060	67,790
sd(NA × (S vs. Q))	0.059	0.044	0.002	0.164	54,199
sd(NA × (WL vs. S))	0.040	0.031	0.002	0.115	72,032
Items					
sd(Intercept)	0.050	0.027	0.003	0.095	4,599
sd(NA)	0.100	0.053	0.006	0.190	4,610
sd(S vs. Q)	0.185	0.064	0.049	0.300	8,825
sd(WL vs. S)	0.150	0.063	0.020	0.258	6,981
sd(NA × (S vs. Q))	0.367	0.128	0.093	0.595	9,005
sd(NA × (WL vs. S))	0.301	0.125	0.040	0.519	7,420

NA: name agreement; WL: word list; S: sentence; Q: quiet.

Models for all dependent variables were run for 24,000 iterations. Bolded values indicate effects where the 95% Cr.I does not contain zero; italicised values indicate effects where the beta estimate is twice the estimate of the standard error.

#### Onset latency

**Figure 4.**
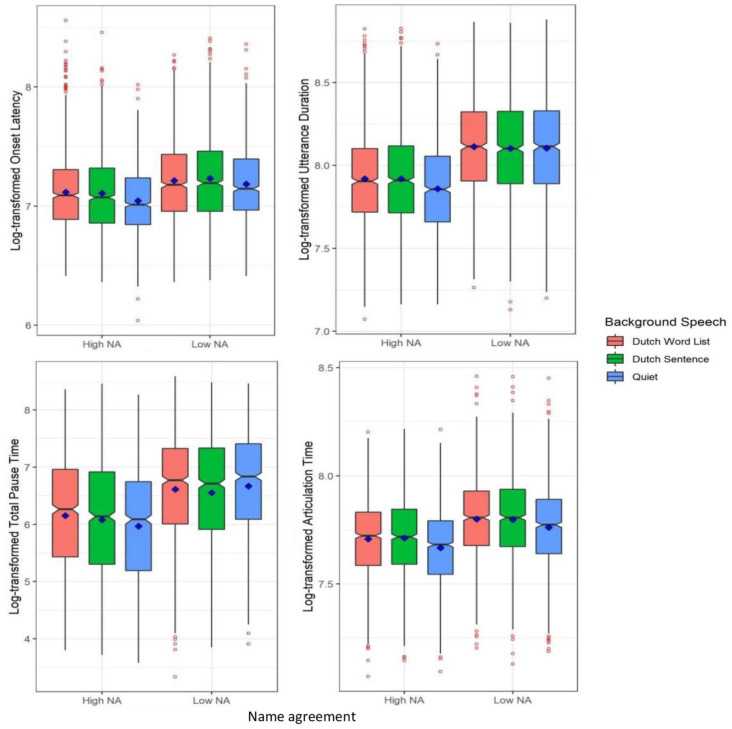
Log-transformed onset latency (top-left), log-transformed utterance duration (top-right), log-transformed total pause time (bottom-left), and log-transformed articulation time (bottom-right) split by name agreement (NA: high, low) and the type of background speech (Dutch word list, Dutch sentence, Quiet) in Experiment 2. Blue squares represent condition means and red points reflect outliers.

As shown in Table 5 and the left panel of Figure 4, a Bayesian mixed-effect model confirmed that log-transformed onset latency was longer when planning names for low name agreement pictures than high name agreement pictures (β = −.128, *SE* = 0.014, 95% Cr.I = [−0.155, −0.1]). There was moderate evidence for the first contrast of background speech (Dutch speech vs. Quiet), such that the log-transformed onset latencies in the two Dutch speech conditions (word list and sentence) were slower than in the quiet condition (β = .076, *SE* = 0.04, 95% Cr.I = [−0.003, 0.155]). While the 95 % Cr.I contains zero, 93% of the posterior distribution around the estimated effect is above zero. Again, name agreement did not interact with the type of background speech.

#### Utterance duration

As shown in Table 5 and the right panel of Figure 4, a Bayesian mixed-effect model showed that the log-transformed utterance duration was longer for low name agreement pictures than high name agreement pictures (β = −.215, *SE* = 0.022, 95% Cr.I = [−0.257, −0.172]). There was moderate evidence for the first contrast of background speech (Dutch speech vs. Quiet), such that the log-transformed utterance durations in the two Dutch speech conditions (word list and sentence) were slower than in the quiet condition (β = .05, *SE* = 0.031, 95% Cr.I = [−0.012, 0.111]). Here, the 95% Cr.I contains zero but 93% of the posterior distribution around the estimated effect is above zero. Again, name agreement did not interact with the type of background speech.

#### Total pause time

As shown in Table 5 and the left panel of Figure 4, a Bayesian mixed-effect model showed that log-transformed total pause time was longer for low name agreement pictures than high name agreement pictures (β = −.599, *SE* = 0.072, 95% Cr.I = [−0.741, −0.458]), but it did not vary with the type of background speech. There was moderate evidence for the interaction of name agreement and the first contrast (Dutch speech vs. Quiet) of background speech (β = .28, *SE* = 0.173, 95% Cr.I = [−0.06, 0.621]). While the 95% Cr.I contains zero, 93% of the posterior distribution around the estimated effect is above zero. This demonstrates that the log-transformed total pause time in the Dutch speech condition was longer than that in the quiet condition for high name agreement pictures (β = .394, *SE* = 0.171, 95% Cr.I = [0.058, 0.727]), but not for low name agreement pictures.

#### Articulation time

As shown in Table 5 and the right panel of Figure 4, a Bayesian mixed-effect model showed that the log-transformed articulation time was affected by both name agreement and the type of background speech: It took longer to articulate names of low name agreement than high name agreement pictures (β = −.093, *SE* = 0.020, 95% Cr.I = [−0.133, −0.054]), and articulation time was longer in the two Dutch speech conditions (word list and sentence) than in the quiet condition (β = .054, *SE* = 0.016, 95% Cr.I = [0.023, 0.085]). There was no interaction between name agreement and the type of background speech.

#### Total chunk number

**Figure 5.**
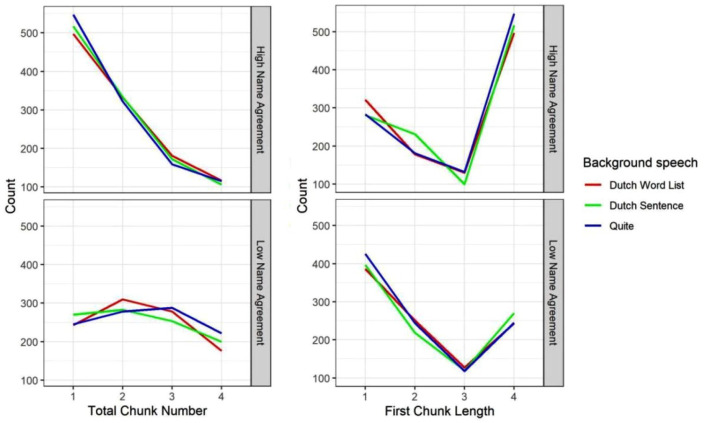
Total chunk number (left) and first chunk length (right) split by name agreement (NA: high, low) and the type of background speech (Dutch word list, Dutch sentence, Quiet) in Experiment 2.

As shown in Table 5 and Figure 5 (left), a Bayesian mixed-effect model showed that participants grouped their responses in more chunks for low name agreement pictures than high name agreement pictures (β = −0.266, *SE* = 0.030, 95% Cr.I = [−0.325, −0.208]). Total chunk number was not impacted by the type of background speech. Again, name agreement did not interact with the type of background speech.

#### First chunk length

As shown in Table 5 and the right panel of Figure 5, a Bayesian mixed-effect model showed that participants planned fewer names in their first response chunk for low name agreement pictures than high name agreement pictures (β = .237, *SE* = 0.027, 95% Cr.I = [0.183, 0.291]). First chunk length was not impacted by the type of background speech. Again, name agreement did not interact with the type of background speech.

### Interim discussion

The results of Experiment 2 were remarkably similar to those of Experiment 1. Consistent with the phonological disruption view (Salamé & Baddeley, 1982, 1989), the presence of background speech, now in the participants’ native language, increased onset latencies and articulation time, and also had a weak impact on utterance durations. There was no difference between the Dutch word list and Dutch sentence conditions on any dependent measures. We also found main effects of name agreement on all dependent measures, and a weak modulation of name agreement on the processing of background speech, such that Dutch background speech increased the total pause time during planning of high, but not low, name agreement pictures. This is consistent with earlier work by He, Meyer and Brehm (2021) and suggests that stronger attentional engagement in the more difficult low name agreement condition leads to less interference from background speech.

## General discussion

In two experiments, we explored how different types of unintelligible (Experiment 1) and intelligible (Experiment 2) background speech affected spoken language production, with a focus on their impact on lexical selection in speech planning. There were four major findings. First, we obtained consistent name agreement effects on all measures in both experiments, with participants producing the names of low name agreement pictures more slowly, with more errors, and in shorter sets (“chunks”) than high name agreement pictures. Second, irrelevant background speech in Experiment 1 (Chinese, unintelligible to speakers) and Experiment 2 (Dutch, intelligible to speakers) always disrupted spoken word production relative to a quiet condition. This patterned as increased articulation time and onset latencies in Experiment 1 (Chinese background speech), and increased articulation time, onset latencies, and utterance duration in Experiment 2 (Dutch background speech). Third, no systematic difference between word lists and sentences was found in either experiment. Finally, there were differences in how the two types of irrelevant speech effects were modulated by the difficulty of speech production: the disruptive effects of Dutch background speech in Experiment 2 were strongest when high name agreement pictures were named.

The effect of name agreement (indexing lexical selection demands in production) was remarkably consistent on all measures and experiments (also see Supplementary Material, Table C1), replicating earlier work (e.g., Alario et al., 2004; He et al., 2021; Shao et al., 2014). The name agreement effects on time measures (onset latencies, utterance duration, total pause time, and articulation time) are noteworthy because they show how the demand of lexical selection affects processing before and after speech onset. This finding suggests that speakers retrieve picture names during the whole process of planning a sequence of picture names, indicative of incremental speech planning during which speakers have to coordinate the planning and articulation of successive words (e.g., Levelt et al., 1999; Roelofs, 1998; Wheeldon & Lahiri, 1997). Moreover, the finding that name agreement affected response chunking measures (total pause time, first chunk length) indicates that increased lexical selection demand reduced planned utterance units in each response, which may reflect that speakers tend to plan names with less temporal overlap, resulting in more and shorter response chunks, for pictures with low, compared with high name agreement.

In both experiments, irrelevant speech consistently increased onset latencies and articulation time relative to a quiet control condition, which is in line with the phonological disruption view (Salamé & Baddeley, 1982, 1989) under the framework of the interference-by-similarity account (e.g., Hughes, 2014; Jones et al., 1993; also the crosstalk account, Pashler, 1994). This view predicts that any background speech (whether it is intelligible or not) should disrupt speech production due to the similarity of phonological codes between the focal task and background speech. Since Dutch speech (Experiment 2) did not cause more disruption than Chinese speech (Experiment 1) during initial planning and articulation processes (see Supplementary Material, Table C1), our results further argue against the importance of semantic similarity in disrupting speech planning.

Combined with earlier results from He, Meyer and Brehm (2021) who showed that word lists (regardless of intelligibility) interfered with onset latencies relative to a speech-like noise condition (i.e., eight-talker babble), these results also argue against the contribution of low-level acoustic properties shared between speech production and speech-like noise. Thus, these results are most in line with the phonological disruption view (Salamé & Baddeley, 1982, 1989).

We also found that Dutch but not Chinese background speech had a weak effect on utterance duration. This is consistent with He, Meyer and Brehm (2021), where Dutch word lists increased utterance duration relative to Chinese word lists, indicating that intelligible background speech elicits more disruption than unintelligible background speech. This suggests that intelligible background speech specifically interferes with the planning that is done between producing chunks of words, where a speaker needs to multi-task between speaking, planning, and listening. The extra disruption on utterance duration may result from similarity in semantics and/or phonology, or from an attention capture mechanism; further research would be needed to disentangle these possibilities.

In contrast to robust differences between background speech and quiet conditions, we did not observe any difference between the background word lists and sentences in either Experiment 1 or 2. The results of Experiment 1 suggest that the stimulus-aspecific variation of unintelligible background speech does not elicit disruption on spoken word production, which goes against the aspecific attention capture view but seems consistent with specific attention capture view (Eimer et al., 1996).

However, the specific attention capture view (Eimer et al., 1996) also predicts that in Experiment 2, Dutch sentences (richer syntactic/semantic representation) should disrupt spoken word production more than Dutch word lists (weaker syntactic/semantic representation). This was not the case: we did not find any difference between Dutch word lists and sentences on any measures in Experiment 2. This is consistent with three possibilities. First, the lack of a word lists versus sentences effect might be because the stimulus-specific effect indeed exists, but it was too small and attenuated by the repetition of stimuli, which all appeared three times across three blocks in the present study. To test this possibility, we conducted all analyses including the repetition (i.e., block) as a within-participant factor. However, we did not find any interaction between the type of irrelevant background speech (word list vs. sentence) and block in either experiment (see Supplementary Material, Table A5 for Experiment 1; Table B3 for Experiment 2), which shows that there is no evidence any background speech effect changes with repetition. Another possibility, and one we deem more likely, is that the aspecific and specific effects may have cancelled each other out. In other words, the disruption by the presence of pauses (aspecific context variation) in Dutch word lists cancelled interference by richer linguistic information (specific linguistic variation) in Dutch sentences. This possibility could be pursued in future research with larger sources of stimulus-specific interference. Finally, it is possible that the manipulation of stimulus-aspecific variation in Experiment 2 was weak because the background speech stimuli were too uniform and boring (word lists had a regular acoustic pattern, sentences had uniform syntactic structure). Participants might adapt to the regular tempo of word lists and use a strategy to name pictures, which causes weaker interference than we predicted and results in the absence of word lists versus sentence effect. This possibility was supported by a follow-up study in He (2023, Chapter 5). This study directly manipulated the relative interestingness (boring vs. funny) of irrelevant background sentences, and found an interestingness effect such that boring sentences were more disruptive than funny sentences. This suggests that stimulus-aspecific variation in the present experiments could have been weak due to the relative uniformity of the stimuli, and also suggests that attention to background speech may be influenced by a wide variety of other factors.

Consistent with the predictions from the attention engagement account (Halin et al., 2014; Marsh et al., 2015), the interaction between background speech and name agreement was absent in Experiment 1 but present in Experiment 2 on the measure of total pause time. Disruption by Chinese background speech remained unaffected by changes in attention engagement manipulated by name agreement because the processing of unintelligible auditory input is automatic and escapes cognitive control (Hughes, 2014). In contrast, interference by Dutch background speech was reduced by increased attention engagement (on low name agreement), because the processing of intelligible background speech requires central attention that taps into cognitive control (Marsh et al., 2018). This is largely consistent with He, Meyer and Brehm (2021), though note that the effects appeared on total pause time in Experiment 2 but on onset latencies in He, Meyer and Brehm 2021. The inconsistency may be due to small effect sizes or to variations in the baseline task (quiet in the present study and eight-talker babble in He, Meyer and Brehm 2021) and the speech production task (naming four pictures in the present study and naming six pictures in He, Meyer and Brehm 2021). Future work is needed to determine the cause of the difference.

The fact that many facets of irrelevant background speech interfere with speech production leaves many possibilities for future work. We sketch some of these now. First, we saw clear evidence for the phonological but not semantic disruption view (Martin et al., 1988; Salamé & Baddeley, 1982, 1989). To understand the nature of interference-by-similarity, more work should therefore be done that considers specific relationships (e.g., phonological, semantic) between speaking and background speech, thereby more cleanly assessing the role of shared representations in speaking-while-listening in a targeted way. Second, this study showed more evidence for specific than aspecific attention capture (Eimer et al., 1996), but could not cleanly distinguish between the two. Future comparisons integrating these two desiderata would be interesting. In particular, a further comparison between different types of irrelevant background speech matched closely on specific content and acoustic variation would be more informative about how two variants of attention capture (aspecific and specific) affect speech production performance in the presence of irrelevant background speech. Third, the non-continuous background speech in this study was regularly timed (with a consistent interval of 700 ms between words), which may have led to habituation effects over time. Future studies with irregular timing in the background speech would provide more clarity regarding the aspecific attention capture account. Fourth, the present research used a multi-object naming task that was relatively easy, and therefore not necessarily representative of typical speech production. Given the complex interplay between the demands of speaking, listening, and attention, it would be fruitful to expand this line of research into more naturalistic speech production tasks such as sentence or dialogue production and to assess whether other aspects of speech production difficulty (such as object recognition, phonological encoding, and phonetic encoding) show similar effects to lexical selection difficulty. Finally, this study mostly focused on the two accounts—the interference-by-similarity and the attention capture account—without considering other theoretical interpretations. Future research should consider alternative explanations for the irrelevant speech effect in speaking. For instance, the timing of the interference could have an effect, based on the results from some PWI studies (e.g., Glaser & Düngelhoff, 1984; Schriefers et al., 1990). This would inform us about other theories for irrelevant speech effect.

## Conclusion

Two experiments using a speaking-while-listening paradigm showed that irrelevant background speech (regardless of its intelligibility) disrupts spoken word production relative to a quiet condition, and that intelligible background speech elicits further disruption. The finding stresses the importance of similarity in phonological representations between the speech production and background speech in eliciting interference. Moreover, the absence of differences between the word list and sentence conditions in unintelligible background speech suggests that the aspecific properties of background speech (in this case, the presence of pauses) do not affect naming performance by diverting attention away from the task. Finally, while intelligible background speech had a larger impact on spoken word production, the impact can be reduced through greater engagement with the task, for example, increasing the difficulty of speech production. The implication is that when the disruption by background speech occurs in speech production, speakers may be able to manage this disruption by changing when and how they plan their speech.

## Supplemental Material

sj-docx-1-qjp-10.1177_17470218231219971 – Supplemental material for Effects of irrelevant unintelligible and intelligible background speech on spoken language production Supplemental material, sj-docx-1-qjp-10.1177_17470218231219971 for Effects of irrelevant unintelligible and intelligible background speech on spoken language production by Jieying He, Candice Frances, Ava Creemers and Laurel Brehm in Quarterly Journal of Experimental Psychology

## Notes

1. Here is an example of Experiment 1 for one participant: https://frinexproduction.mpi.nl/image_naming_noise_cn/?stimulusList=List1.

2. This was a different speaker from the one who recorded Dutch words for attention check trials.

3. Here is an example of Experiment 2 for one participant: https://frinexproduction.mpi.nl/image_naming_noise_nl/?stimulusList=List1.
