# Supplementary material for: Effects of irrelevant unintelligible and intelligible background speech on spoken language production
Source: Q J Exp Psychol (Hove). 2024 Jan 21;77(8):1745–69. doi: 10.1177/17470218231219971 (PMC11295403; doi:10.1177/17470218231219971)
Supplement: sj-docx-1-qjp-10.1177_17470218231219971 – Supplemental material for Effects of irrelevant unintelligible and intelligible background speech on spoken language production [file sj-docx-1-qjp-10.1177_17470218231219971.docx]

**Supplementary Material for**

# Effects of irrelevant unintelligible and intelligible background speech on spoken language production

Jieying He^a,b*^, Candice Frances^a^, Ava Creemers ^a,^, Laurel Brehm^a,c^

^a^ Max Planck Institute for Psycholinguistics, Nijmegen, The Netherlands

^b^ International Max Planck Research School for Language Sciences, Nijmegen, The Netherlands

^c^ Department of Linguistics, University of California, Santa Barbara, California, USA

^*^ Correspondence concerning this article should be addressed to Jieying He, Max Planck Institute for Psycholinguistics, P.O. Box 310, 6500 AH Nijmegen, The Netherlands. Email: [Jieying.He@mpi.nl](mailto:Jieying.He@mpi.nl)

***A. Supplementary Material* *for Experiment 1.***

Table A1. 240 pictures used in both Experiments.

| *Picture Grid* | *Picture 1* | *Picture 2* | *Picture 3* | *Picture 4* |  | *Picture Grid* | *Picture 1* | *Picture 2* | *Picture 3* | *Picture 4* |
| --- | --- | --- | --- | --- | --- | --- | --- | --- | --- | --- |
| *Pictures with high name agreement* | | | | | | | | | | |
| 1 | koelkast | pijl | dolfijn | gevangenis |  | 16 | spiegel | ananas | robot | zaklamp |
| 2 | leeuw | kruiwagen | driehoek | tomaat |  | 17 | schilderij | tunnel | kangoeroe | broek |
| 3 | harp | radio | knie | paprika |  | 18 | sleutel | dobbelsteen | ketting | rechter |
| 4 | vlinder | trap | cactus | batterij |  | 19 | stopcontact | arm | ezel | diamant |
| 5 | zaag | kiwi | vliegtuig | bezem |  | 20 | kapper | zebra | aardbei | wolk |
| 6 | waaier | schaap | glas | baard |  | 21 | schaduw | kompas | geit | horloge |
| 7 | ster | konijn | doedelzak | handschoen |  | 22 | pompoen | vlieger | kaars | skelet |
| 8 | pijp | hamer | berg | duim |  | 23 | heks | aardappel | vleermuis | boog |
| 9 | eekhoorn | keuken | banaan | orkest |  | 24 | masker | bijbel | zwembad | kanon |
| 10 | kwal | slager | anker | vuist |  | 25 | schaar | rups | kraan | puzzel |
| 11 | microfoon | bloem | koning | stier |  | 26 | eiland | schildpad | clown | bril |
| 12 | kokosnoot | steen | gitaar | egel |  | 27 | fruit | vlag | aansteker | lepel |
| 13 | roos | trechter | kroon | ballon |  | 28 | kikker | wasmachine | bokser | trompet |
| 14 | slak | rug | weegschaal | honing |  | 29 | bus | fabriek | sok | vork |
| 15 | muis | drumstel | parachute | tandarts |  | 30 | papegaai | helikopter | toetsenbord | riem |
| *Pictures with low name agreement* | | | | | | | | | | |
| 1 | jager | klauw | baksteen | trui |  | 16 | antenne | olie | piano | knuffel |
| 2 | lade | schedel | melk | foto |  | 17 | planeet | motor | litteken | gang |
| 3 | speer | nagel | kerkhof | duif |  | 18 | komkommer | badkamer | domino | wortels |
| 4 | engel | parel | troon | viool |  | 19 | schatkist | elf | koffie | put |
| 5 | kasteel | snoepje | brievenbus | vogelkooi |  | 20 | schelp | prullenbak | ridder | meloen |
| 6 | kerk | schoolbord | bank | walrus |  | 21 | hengel | gevangene | brug | driewieler |
| 7 | soldaat | vis | gorilla | kruk |  | 22 | vinger | magneet | zanger | plas |
| 8 | armband | rimpels | kogel | hagedis |  | 23 | blad | raam | jurk | hoorn |
| 9 | ijsje | spuit | paus | badkuip |  | 24 | rivier | monster | pion | goochelaar |
| 10 | broekzak | naald | varken | wasbak |  | 25 | rugzak | chocolade | balkon | schep |
| 11 | staart | inktvis | herder | perzik |  | 26 | koekje | garage | cirkel | mossel |
| 12 | sigaret | ijsberg | hersenen | kwast |  | 27 | camping | pruik | sneeuw | ballerina |
| 13 | gymzaal | leraar | handdoek | worst |  | 28 | munt | strand | kameel | lamp |
| 14 | museum | tuinslang | druif | kegel |  | 29 | kleed | tram | doodskist | garnaal |
| 15 | koningin | buik | trein | soep |  | 30 | haven | bliksem | schrift | kaarten |

Table A2. 20 Chinese word lists used in Experiment 1.

|  | *Noun 1* | *Noun 2* | *Noun 3* | *Noun 4* | *Noun 5* | *Noun 6* |
| --- | --- | --- | --- | --- | --- | --- |
| List1 | 剑 | 苍蝇 | 梨 | 画家 | 暖气 | 幸运草 |
| List2 | 肉 | 火箭 | 羽毛 | 鞋带 | 正方形 | 树枝 |
| List3 | 美洲豹 | 邮票 | 胸 | 电视 | 剃刀 | 发梳 |
| List4 | 奶酪 | 枭 | 植物 | 救护车 | 眼睛 | 手鼓 |
| List5 | 老鹰 | 火 | 风扇 | 纽扣 | 鼓 | 摄影师 |
| List6 | 巢 | 早餐 | 樵夫 | 屁股 | 立方体 | 铁刷 |
| List7 | 鸟 | 船舵 | 刽子手 | 嘴唇 | 温室 | 步枪 |
| List8 | 手风琴 | 肩膀 | 秃鹫 | 鞋 | 衣柜 | 骨头 |
| List9 | 肺 | 盆子 | 栅栏 | 计算器 | 迷宫 | 蛇 |
| List10 | 仙女 | 奖章 | 船 | 秃头 | 桌子 | 面包机 |
| List11 | 树 | 火山 | 袋子 | 磨坊 | 鳄鱼 | 洋娃娃 |
| List12 | 波浪 | 橄榄 | 钉子 | 相机 | 音乐会 | 鹅 |
| List13 | 机场 | 杯子 | 肥皂 | 狼 | 盒子 | 向日葵 |
| List14 | 血管 | 帽子 | 文件夹 | 河马 | 烟 | 豆子 |
| List15 | 橡子 | 游泳者 | 盘子 | 钱包 | 鸡 | 眉毛 |
| List16 | 独木舟 | 戒指 | 西瓜 | 马 | 公主 | 椅子 |
| List17 | 渔夫 | 合唱团 | 足球 | 苹果 | 超市 | 鹿 |
| List18 | 瓶塞 | 灭火器 | 柠檬 | 香水 | 铅笔 | 锁 |
| List19 | 盐 | 坦克 | 奶牛 | 服务员 | 黄金 | 床垫 |
| List20 | 裙子 | 电缆 | 脚 | 摇篮 | 护士 | 水族馆 |

Table A3. 20 Chinese sentences used in Experiment 1.

| No. | Chinese sentences |
| --- | --- |
| 1 | 幸运草和暖气在画家的左边，并且梨和苍蝇在剑的右边。 |
| 2 | 树枝和正方形在鞋带的左边，并且羽毛和火箭在肉的左边。 |
| 3 | 发梳和剃刀在电视的右边，并且胸和邮票在美洲豹的左边。 |
| 4 | 手鼓和眼睛在救护车的右边，并且植物和枭在奶酪的右边。 |
| 5 | 摄影师和鼓在纽扣的左边，并且风扇和火在老鹰的右边。 |
| 6 | 铁刷和立方体在屁股的左边，并且樵夫和早餐在巢的左边。 |
| 7 | 步枪和温室在嘴唇的右边，并且刽子手和船舵在鸟的左边。 |
| 8 | 骨头和衣柜在鞋的右边，并且秃鹫和肩膀在手风琴的右边。 |
| 9 | 蛇和迷宫在计算器的左边，并且栅栏和盆子在肺的右边。 |
| 10 | 面包机和桌子在秃头的左边，并且船和奖章在仙女的左边。 |
| 11 | 洋娃娃和鳄鱼在磨坊的右边，并且袋子和火山在树的左边。 |
| 12 | 鹅和音乐会在相机的右边，并且钉子和橄榄在波浪的右边。 |
| 13 | 向日葵和盒子在狼的左边，并且肥皂和杯子在机场的右边。 |
| 14 | 豆子和烟在河马的左边，并且文件夹和帽子在血管的左边。 |
| 15 | 眉毛和鸡在钱包的右边，并且盘子和游泳者在橡子左边。 |
| 16 | 椅子和公主在马的右边，并且西瓜和戒指在独木舟的右边。 |
| 17 | 鹿和超市在苹果的左边，并且足球和合唱团在渔夫的右边。 |
| 18 | 锁和铅笔在香水的左边，并且柠檬和灭火器在瓶塞的左边。 |
| 19 | 床垫和黄金在服务员右边，并且奶牛和坦克在盐的左边。 |
| 20 | 水族馆和护士在摇篮的右边，并且脚和电缆在裙子的右边。 |

Table A4. A questionnaire of Chinese experience in Experiment 1.

| Tot slot willen we je vragen om een aantal vragen te beantwoorden over jouw ervaring met Mandarijn Chinees. Nadat je een vraag hebt aangevinkt, dien je op ‘Volgende’ te klikken om naar de volgende vraag te gaan. |
| --- |
| 1) Ben je in een land geweest waar Mandarijn Chinees wordt gesproken? Zo ja, hoeveel maanden?  A. Nooit B. <3 maanden C. 3-6 maanden D. 6-12 maanden E. >12 maanden |
| 2) Ben je bij een gezin geweest waar Mandarijn Chinees wordt gesproken? Zo ja, hoeveel maanden?  A. Nooit B. <3 maanden C. 3-6 maanden D. 6-12 maanden E. >12 maanden |
| 3) Ben je in een school/werkomgeving geweest waar Mandarijn Chinees wordt gesproken? Zo ja, hoeveel maanden?  A. Nooit B. <3 maanden C. 3-6 maanden D. 6-12 maanden E. >12 maanden |
| 4) Gebruik onderstaande schaal, waar 0 “helemaal geen kennis” is, en 10 “vloeiend, alsof het je moedertaal is”. Geef aan wat jouw vaardigheidsniveau is op het gebied van het spreken, verstaan en lezen van Mandarijn Chinees.  A. Spreken van Mandarijn Chinees: 0 1 2 3 4 5 6 7 8 9 10  B. Verstaan van gesproken Mandarijn Chinees: 0 1 2 3 4 5 6 7 8 9 10  C. Lezen van Mandarijn Chinees: 0 1 2 3 4 5 6 7 8 9 10 |
| 5) Gebruik onderstaande schaal, waar 0 “helemaal geen kennis” is, en 10 “vloeiend, alsof het je moedertaal is”. Geef aan in hoeverre je op dit moment blootgesteld wordt aan Mandarijn Chinees in de volgende situaties.  A. Contact hebben met Chinese vrienden: 0 1 2 3 4 5 6 7 8 9 10  B. Kijken van Chinese TV: 0 1 2 3 4 5 6 7 8 9 10  C. Luisteren naar Chinese radio/muziek: 0 1 2 3 4 5 6 7 8 9 10  D. Lezen van Chinese boeken/tijdschriften: 0 1 2 3 4 5 6 7 8 9 10 |

Table A5. Results of block analysis in Experiment 1.

|  |  | Estimate | Est.error | 95% Cr. I | | Effective samples |
| --- | --- | --- | --- | --- | --- | --- |
|  |  |  |  | lower | upper |  |
| ***Log-transformed onset latency*** | | | | | | |
| *Population-level effects* | Intercept | 7.134 | 0.028 | 7.079 | 7.19 | 5611 |
|  | Name Agreement | **-0.121** | **0.015** | **-0.15** | **-0.092** | **60182** |
|  | Speech vs. Quiet | **0.062** | **0.024** | **0.015** | **0.11** | **59671** |
|  | Word List vs. Sentence | 0 | 0.021 | -0.041 | 0.041 | 62160 |
|  | Block 12 vs. Block 3 | **0.194** | **0.029** | **0.136** | **0.25** | **51032** |
|  | Block 1 vs. Block 2 | **0.245** | **0.028** | **0.19** | **0.299** | **42710** |
|  | NA × (S vs. Q) | -0.004 | 0.042 | -0.086 | 0.079 | 66494 |
|  | NA × (WL vs. S) | -0.019 | 0.039 | -0.096 | 0.059 | 68857 |
|  | NA × (Block 12 vs. 3) | -0.035 | 0.046 | -0.125 | 0.055 | 69736 |
|  | NA × (Block 1 vs. 2) | -0.01 | 0.037 | -0.083 | 0.062 | 66348 |
|  | (S vs. Q) × (Block 12 vs. 3) | 0.026 | 0.051 | -0.074 | 0.126 | 74295 |
|  | (WL vs. S) × (Block 12 vs. 3) | -0.023 | 0.049 | -0.12 | 0.075 | 67668 |
|  | (S vs. Q) × (Block 1 vs. 2) | 0.093 | 0.047 | 0 | 0.185 | 65723 |
|  | (WL vs. S) × (Block 1 vs. 2) | -0.029 | 0.055 | -0.136 | 0.078 | 70992 |
|  | NA × (S vs. Q) × (Block 12vs.3) | 0.047 | 0.095 | -0.138 | 0.233 | 77572 |
|  | NA × (WL vs. S) × (Block 12vs.3) | 0.025 | 0.087 | -0.146 | 0.194 | 82091 |
|  | NA × (S vs. Q) × (Block 1vs.2) | -0.017 | 0.082 | -0.179 | 0.146 | 79468 |
|  | NA × (WL vs. S) × (Block 1vs.2) | -0.013 | 0.098 | -0.205 | 0.18 | 76734 |
|  | | | | | | |
| ***Log-transformed utterance duration*** | | | | | | |
| *Population-level effects* | Intercept | 8.021 | 0.023 | 7.975 | 8.067 | 6748 |
|  | Name Agreement | **-0.191** | **0.02** | **-0.23** | **-0.151** | **52806** |
|  | Speech vs. Quiet | **0.03** | **0.012** | **0.006** | **0.054** | **85083** |
|  | Word List vs. Sentence | -0.003 | 0.011 | -0.025 | 0.019 | 87020 |
|  | Block 12 vs. Block 3 | **0.168** | **0.019** | **0.132** | **0.205** | **49646** |
|  | Block 1 vs. Block 2 | **0.134** | **0.016** | **0.103** | **0.166** | **46638** |
|  | NA × (S vs. Q) | 0.015 | 0.024 | -0.031 | 0.062 | 90001 |
|  | NA × (WL vs. S) | 0.005 | 0.023 | -0.041 | 0.051 | 80784 |
|  | NA × (Block 12 vs. 3) | **-0.101** | **0.025** | **-0.149** | **-0.052** | **87321** |
|  | NA × (Block 1 vs. 2) | **-0.073** | **0.024** | **-0.12** | **-0.026** | **82973** |
|  | (S vs. Q) × (Block 12 vs. 3) | -0.022 | 0.053 | -0.125 | 0.083 | 63183 |
|  | (WL vs. S) × (Block 12 vs. 3) | -0.066 | 0.046 | -0.156 | 0.025 | 65632 |
|  | (S vs. Q) × (Block 1 vs. 2) | 0.031 | 0.049 | -0.066 | 0.127 | 64491 |
|  | (WL vs. S) × (Block 1 vs. 2) | -0.029 | 0.04 | -0.107 | 0.049 | 61714 |
|  | NA × (S vs. Q) × (Block 12vs.3) | 0.033 | 0.096 | -0.156 | 0.22 | 69797 |
|  | NA × (WL vs. S) × (Block 12vs.3) | -0.005 | 0.085 | -0.171 | 0.163 | 73221 |
|  | NA × (S vs. Q) × (Block 1vs.2) | -0.048 | 0.09 | -0.224 | 0.129 | 74468 |
|  | NA × (WL vs. S) × (Block 1vs.2) | 0.03 | 0.073 | -0.113 | 0.173 | 69539 |
|  | | | | | | |
| ***Log-transformed total pause time*** | | | | | | |
| *Population-level effects* | Intercept | 5.019 | 0.291 | 4.447 | 5.59 | 4615 |
|  | Name Agreement | **-1.429** | **0.241** | **-1.904** | **-0.952** | **14775** |
|  | Speech vs. Quiet | -0.428 | 0.238 | -0.896 | 0.037 | 37330 |
|  | Word List vs. Sentence | -0.115 | 0.2 | -0.505 | 0.278 | 45039 |
|  | Block 12 vs. Block 3 | **1.131** | **0.22** | **0.699** | **1.562** | **29293** |
|  | Block 1 vs. Block 2 | **0.912** | **0.18** | **0.558** | **1.263** | **28534** |
|  | NA × (S vs. Q) | -0.023 | 0.365 | -0.74 | 0.7 | 68037 |
|  | NA × (WL vs. S) | 0.137 | 0.348 | -0.546 | 0.819 | 55847 |
|  | NA × (Block 12 vs. 3) | -0.05 | 0.419 | -0.871 | 0.779 | 54403 |
|  | NA × (Block 1 vs. 2) | -0.214 | 0.302 | -0.808 | 0.378 | 70396 |
|  | (S vs. Q) × (Block 12 vs. 3) | 0.569 | 0.564 | -0.544 | 1.676 | 57132 |
|  | (WL vs. S) × (Block 12 vs. 3) | -0.252 | 0.566 | -1.361 | 0.864 | 55234 |
|  | (S vs. Q) × (Block 1 vs. 2) | 0.118 | 0.475 | -0.813 | 1.048 | 59261 |
|  | (WL vs. S) × (Block 1 vs. 2) | 0.578 | 0.449 | -0.309 | 1.458 | 55047 |
|  | NA × (S vs. Q) × (Block 12vs.3) | 1.233 | 1.129 | -0.994 | 3.441 | 48396 |
|  | NA × (WL vs. S) × (Block 12vs.3) | -0.12 | 1.101 | -2.281 | 2.031 | 56935 |
|  | NA × (S vs. Q) × (Block 1vs.2) | -0.75 | 0.935 | -2.586 | 1.093 | 63045 |
|  | NA × (WL vs. S) × (Block 1vs.2) | 0.981 | 0.818 | -0.619 | 2.586 | 59252 |
|  |  |  |  |  |  |  |

*Note.* NA refers to name agreement, WL refers to word lists, S refers to sentences. These results are for 36 participants who wore their headphones/earphones correctly.

***B. Supplementary Material for Experiment 2***

Table B1. 20 Dutch word lists used in Experiment 2.

|  | *Noun 1* | *Noun 2* | *Noun 3* | *Noun 4* | *Noun 5* | *Noun 6* |
| --- | --- | --- | --- | --- | --- | --- |
| List1 | fee | medaille | boot | luipaard | zonnebloem | kers |
| List2 | tak | beker | prinses | schild | veer | raket |
| List3 | postzegel | vlees | jas | tamboerijn | map | kam |
| List4 | plant | Kaas | accordeon | oog | scheermes | uil |
| List5 | rekenmachine | mand | vulkaan | zeep | paard | kano |
| List6 | gier | vierkant | schoen | ambulance | kast | boom |
| List7 | krokodil | veter | tas | molen | pop | bot |
| List8 | ring | slang | dienblad | hek | watermeloen | kubus |
| List9 | nest | ontbijt | borstel | trommel | stoel | kruik |
| List10 | potlood | Kurk | brandblusser | citroen | spons | vuur |
| List11 | nijlpaard | koffer | spijker | camera | fakkel | boon |
| List12 | vliegveld | Wolf | kopje | houthakker | doos | boter |
| List13 | televisie | zwaard | voet | peer | schilder | klavertje |
| List14 | vlieg | Rok | zuster | kabel | aquarium | wieg |
| List15 | zwemmer | Lijst | bord | portemonnee | hert | koor |
| List16 | ventilator | Zout | adelaar | tank | liniaal | brief |
| List17 | koe | voetbal | goud | wortel | parfum | serveerster |
| List18 | kas | Gans | tafel | verwarming | fotograaf | roer |
| List19 | appel | theepot | knoop | vogel | wandelstok | slot |
| List20 | pet | cadeau | haak | olijf | kip | visser |

Table B2. 20 Dutch sentences used in Experiment 2.

| *No.* | *Dutch sentences* |
| --- | --- |
| 1 | De kers en de zonnebloem bevinden zich links van het luipaard, en de boot en de medaille bevinden zich rechts van de fee. |
| 2 | De raket en de veer bevinden zich links van het schild, en de prinses en de beker bevinden zich links van de tak. |
| 3 | De kam en de map bevinden zich rechts van de tamboerijn, en de jas en het vlees bevinden zich links van de postzegel. |
| 4 | De uil en het scheermes bevinden zich rechts van het oog, en de accordeon en de kaas bevinden zich rechts van de plant. |
| 5 | De kano en het paard bevinden zich links van de zeep, en de vulkaan en de mand bevinden zich rechts van de rekenmachine. |
| 6 | De boom en de kast bevinden zich links van de ambulance, en de schoen en het vierkant bevinden zich links van de gier. |
| 7 | Het bot en de pop bevinden zich rechts van de molen, en de tas en de veter bevinden zich links van de krokodil. |
| 8 | De kubus en de watermeloen bevinden zich rechts van het hek, en het dienblad en de slang bevinden zich rechts van de ring. |
| 9 | De kruik en de stoel bevinden zich links van de trommel, en de borstel en het ontbijt bevinden zich rechts van het nest. |
| 10 | Het vuur en de spons bevinden zich links van de citroen, en de brandblusser en de kurk bevinden zich links van het potlood. |
| 11 | De boon en de fakkel bevinden zich rechts van de camera, en de spijker en de koffer bevinden zich links van het nijlpaard. |
| 12 | De boter en de doos bevinden zich rechts van de houthakker, en het kopje en de wolf bevinden zich rechts van het vliegveld. |
| 13 | Het klavertje en de schilder bevinden zich links van de peer, en de voet en het zwaard bevinden zich rechts van de televisie. |
| 14 | De wieg en het aquarium bevinden zich links van de kabel, en de zuster en de rok bevinden zich links van de vlieg. |
| 15 | Het koor en het hert bevinden zich rechts van de portemonnee, en het bord en de lijst bevinden zich links van de zwemmer. |
| 16 | De brief en de liniaal bevinden zich rechts van de tank, en de adelaar en het zout bevinden zich rechts van de ventilator. |
| 17 | De serveerster en het parfum bevinden zich links van de wortel, en het goud en de voetbal bevinden zich rechts van de koe. |
| 18 | Het roer en de fotograaf bevinden zich links van de verwarming, en de tafel en de gans bevinden zich links van de kas. |
| 19 | Het slot en de wandelstok bevinden zich rechts van de vogel, en de knoop en de theepot bevinden zich links van de appel. |
| 20 | De visser en de kip bevinden zich rechts van de olijf, en de haak en het cadeau bevinden zich rechts van de pet. |

Table B3. Results of block analysis in Experiment 2.

|  |  | Estimate | Est.error | 95% Cr. I | | Effective samples |
| --- | --- | --- | --- | --- | --- | --- |
|  |  |  |  | lower | upper |  |
| ***Log-transformed onset latency*** | | | | | | |
| *Population-level effects* | Intercept | 7.161 | 0.028 | 7.106 | 7.217 | 4693 |
|  | Name Agreement | **-0.127** | **0.013** | **-0.153** | **-0.101** | **56007** |
|  | Speech vs. Quiet | **0.076** | **0.022** | **0.033** | **0.119** | **55853** |
|  | Word List vs. Sentence | -0.005 | 0.019 | -0.043 | 0.033 | 59827 |
|  | Block 12 vs. Block 3 | **0.236** | **0.027** | **0.183** | **0.288** | **36045** |
|  | Block 1 vs. Block 2 | **0.301** | **0.028** | **0.246** | **0.356** | **35931** |
|  | NA × (S vs. Q) | 0.043 | 0.039 | -0.034 | 0.121 | 60049 |
|  | NA × (WL vs. S) | 0.029 | 0.036 | -0.043 | 0.1 | 61253 |
|  | NA × (Block 12 vs. 3) | -0.06 | 0.038 | -0.136 | 0.014 | 61001 |
|  | NA × (Block 1 vs. 2) | -0.064 | 0.037 | -0.137 | 0.009 | 62117 |
|  | (S vs. Q) × (Block 12 vs. 3) | 0.074 | 0.051 | -0.026 | 0.175 | 63417 |
|  | (WL vs. S) × (Block 12 vs. 3) | -0.01 | 0.043 | -0.095 | 0.075 | 62381 |
|  | (S vs. Q) × (Block 1 vs. 2) | **0.221** | **0.048** | **0.126** | **0.315** | **56880** |
|  | (WL vs. S) × (Block 1 vs. 2) | -0.045 | 0.046 | -0.137 | 0.047 | 61468 |
|  | NA × (S vs. Q) × (Block 12vs.3) | -0.014 | 0.091 | -0.19 | 0.165 | 68028 |
|  | NA × (WL vs. S) × (Block 12vs.3) | -0.046 | 0.081 | -0.205 | 0.115 | 67893 |
|  | NA × (S vs. Q) × (Block 1vs.2) | -0.11 | 0.084 | -0.274 | 0.056 | 70312 |
|  | NA × (WL vs. S) × (Block 1vs.2) | -0.024 | 0.086 | -0.193 | 0.145 | 66811 |
|  | | | | | | |
| ***Log-transformed utterance duration*** | | | | | | |
| *Population-level effects* | Intercept | 8.012 | 0.028 | 7.957 | 8.067 | 4964 |
|  | Name Agreement | **-0.214** | **0.022** | **-0.256** | **-0.171** | **36308** |
|  | Speech vs. Quiet | **0.05** | **0.015** | **0.02** | **0.081** | **56830** |
|  | Word List vs. Sentence | 0.004 | 0.011 | -0.018 | 0.027 | 72507 |
|  | Block 12 vs. Block 3 | **0.189** | **0.018** | **0.153** | **0.225** | **34819** |
|  | Block 1 vs. Block 2 | **0.16** | **0.015** | **0.131** | **0.19** | **52287** |
|  | NA × (S vs. Q) | **0.073** | **0.028** | **0.018** | **0.128** | **65023** |
|  | NA × (WL vs. S) | -0.007 | 0.023 | -0.051 | 0.038 | 69775 |
|  | NA × (Block 12 vs. 3) | **-0.095** | **0.026** | **-0.146** | **-0.045** | **70942** |
|  | NA × (Block 1 vs. 2) | **-0.063** | **0.025** | **-0.112** | **-0.014** | **65090** |
|  | (S vs. Q) × (Block 12 vs. 3) | -0.061 | 0.056 | -0.17 | 0.049 | 50549 |
|  | (WL vs. S) × (Block 12 vs. 3) | -0.05 | 0.051 | -0.15 | 0.051 | 48181 |
|  | (S vs. Q) × (Block 1 vs. 2) | 0.014 | 0.049 | -0.082 | 0.109 | 47859 |
|  | (WL vs. S) × (Block 1 vs. 2) | -0.021 | 0.044 | -0.108 | 0.066 | 50218 |
|  | NA × (S vs. Q) × (Block 12vs.3) | 0.097 | 0.096 | -0.093 | 0.285 | 58207 |
|  | NA × (WL vs. S) × (Block 12vs.3) | 0.096 | 0.09 | -0.082 | 0.272 | 57433 |
|  | NA × (S vs. Q) × (Block 1vs.2) | 0.052 | 0.089 | -0.123 | 0.226 | 56100 |
|  | NA × (WL vs. S) × (Block 1vs.2) | 0.066 | 0.08 | -0.092 | 0.224 | 57018 |
|  | | | | | | |
| ***Log-transformed total pause time*** | | | | | | |
| *Population-level effects* | Intercept | 6.294 | 0.088 | 6.121 | 6.468 | 6219 |
|  | Name Agreement | **-0.598** | **0.073** | **-0.741** | **-0.454** | **37565** |
|  | Speech vs. Quiet | 0.052 | 0.053 | -0.052 | 0.156 | 74627 |
|  | Word List vs. Sentence | 0.055 | 0.046 | -0.036 | 0.146 | 77117 |
|  | Block 12 vs. Block 3 | **0.475** | **0.07** | **0.338** | **0.612** | **40543** |
|  | Block 1 vs. Block 2 | **0.413** | **0.06** | **0.295** | **0.531** | **50115** |
|  | NA × (S vs. Q) | **0.292** | **0.111** | **0.075** | **0.512** | **72640** |
|  | NA × (WL vs. S) | -0.017 | 0.094 | -0.202 | 0.167 | 78343 |
|  | NA × (Block 12 vs. 3) | **-0.27** | **0.101** | **-0.469** | **-0.07** | **77865** |
|  | NA × (Block 1 vs. 2) | -0.138 | 0.097 | -0.331 | 0.053 | 72022 |
|  | (S vs. Q) × (Block 12 vs. 3) | -0.041 | 0.185 | -0.405 | 0.322 | 61523 |
|  | (WL vs. S) × (Block 12 vs. 3) | -0.03 | 0.173 | -0.369 | 0.312 | 60175 |
|  | (S vs. Q) × (Block 1 vs. 2) | -0.046 | 0.175 | -0.389 | 0.296 | 56617 |
|  | (WL vs. S) × (Block 1 vs. 2) | 0.106 | 0.15 | -0.189 | 0.402 | 57255 |
|  | NA × (S vs. Q) × (Block 12vs.3) | 0.324 | 0.35 | -0.364 | 1.013 | 67276 |
|  | NA × (WL vs. S) × (Block 12vs.3) | 0.482 | 0.335 | -0.179 | 1.136 | 64208 |
|  | NA × (S vs. Q) × (Block 1vs.2) | 0.215 | 0.308 | -0.388 | 0.821 | 63082 |
|  | NA × (WL vs. S) × (Block 1vs.2) | 0.256 | 0.285 | -0.306 | 0.816 | 64384 |

*Note.* NA refers to name agreement, WL refers to word lists, S refers to sentences. These results are for 36 participants who wore their headphones/earphones correctly.

***C. Supplementary Material: Comparison of two experiments***

Table C1. Results of Bayesian mixed-effect models across experiments.

|  |  | Estimate | Est.error | 95% Cr. I | | Effective samples |
| --- | --- | --- | --- | --- | --- | --- |
|  |  |  |  | lower | upper |  |
| ***Log-transformed onset latency*** | | | | | | |
| *Population-level effects* | Intercept | 7.147 | 0.019 | 7.11 | 7.186 | 5824 |
|  | Name Agreement | **-0.125** | **0.012** | **-0.149** | **-0.101** | **63985** |
|  | Speech vs. Quiet | **0.07** | **0.036** | **0** | **0.141** | **71154** |
|  | Word List vs. Sentence | -0.003 | 0.04 | -0.081 | 0.075 | 68553 |
|  | Experiment | -0.026 | 0.037 | -0.098 | 0.046 | 6025 |
|  | NA × (S vs. Q) | 0.017 | 0.068 | -0.117 | 0.15 | 71792 |
|  | NA × (WL vs. S) | 0.005 | 0.074 | -0.142 | 0.15 | 70402 |
|  | NA × Experiment | 0.005 | 0.013 | -0.021 | 0.031 | 70888 |
|  | (S vs. Q) × Experiment | -0.013 | 0.032 | -0.076 | 0.05 | 74191 |
|  | (WL vs. S) × Experiment | 0.003 | 0.029 | -0.054 | 0.06 | 72758 |
|  | NA × (S vs. Q) × Experiment | -0.049 | 0.056 | -0.158 | 0.059 | 75539 |
|  | NA × (WL vs. S) × Experiment | -0.039 | 0.054 | -0.145 | 0.067 | 75976 |
|  |  |  |  |  |  |  |
| *Group-level effects* | *Participant_sd (Intercept)* | 0.17 | 0.014 | 0.146 | 0.199 | 10874 |
|  | sd(Name Agreement) | 0.027 | 0.008 | 0.01 | 0.041 | 22835 |
|  | sd(Speech vs. Quiet) | 0.065 | 0.01 | 0.047 | 0.084 | 36544 |
|  | sd(Word List vs. Sentence) | 0.04 | 0.009 | 0.021 | 0.058 | 20658 |
|  | sd(NA × (S vs. Q)) | 0.025 | 0.017 | 0.001 | 0.064 | 28855 |
|  | sd(NA × (WL vs. S)) | 0.021 | 0.016 | 0.001 | 0.059 | 26258 |
|  | *Item_sd (Intercept)* | 0.027 | 0.013 | 0.002 | 0.048 | 1450 |
|  | sd(Name Agreement) | 0.055 | 0.026 | 0.004 | 0.096 | 1385 |
|  | sd(Speech vs. Quiet) | 0.167 | 0.095 | 0.007 | 0.307 | 1211 |
|  | sd(Word List vs. Sentence) | 0.192 | 0.105 | 0.009 | 0.344 | 1842 |
|  | sd(Experiment) | 0.018 | 0.011 | 0.001 | 0.038 | 2045 |
|  | sd(NA × (S vs. Q)) | 0.347 | 0.189 | 0.016 | 0.616 | 1209 |
|  | sd(NA × (WL vs. S)) | 0.381 | 0.211 | 0.016 | 0.687 | 1817 |
|  | sd(NA × Experiment) | 0.037 | 0.021 | 0.002 | 0.075 | 1954 |
|  | sd( (S vs. Q) × Experiment) | 0.124 | 0.07 | 0.006 | 0.23 | 1526 |
|  | sd( (WL vs. S) × Experiment) | 0.131 | 0.058 | 0.012 | 0.225 | 3159 |
|  | sd(NA × (S vs. Q) × Experiment) | 0.25 | 0.14 | 0.011 | 0.461 | 1548 |
|  | sd(NA × (WL vs. S) × Experiment) | 0.258 | 0.117 | 0.023 | 0.446 | 3247 |
|  | | | | | | |
| ***Log-transformed utterance duration*** | | | | | | |
| *Population-level effects* | Intercept | 8.016 | 0.019 | 7.979 | 8.053 | 4034 |
|  | Name Agreement | **-0.204** | **0.019** | **-0.24** | **-0.166** | **31359** |
|  | Speech vs. Quiet | 0.039 | 0.027 | -0.014 | 0.093 | 38700 |
|  | Word List vs. Sentence | 0.001 | 0.022 | -0.043 | 0.045 | 37920 |
|  | Experiment | 0.01 | 0.033 | -0.054 | 0.075 | 3561 |
|  | NA × (S vs. Q) | 0.045 | 0.053 | -0.06 | 0.149 | 39293 |
|  | NA × (WL vs. S) | -0.001 | 0.044 | -0.087 | 0.085 | 38949 |
|  | NA × Experiment | 0.024 | 0.018 | -0.011 | 0.059 | 21478 |
|  | (S vs. Q) × Experiment | *-0.02* | *0.015* | *-0.05* | *0.009* | *62382* |
|  | (WL vs. S) × Experiment | -0.007 | 0.013 | -0.032 | 0.017 | 69948 |
|  | NA × (S vs. Q) × Experiment | **-0.055** | **0.027** | **-0.109** | **-0.001** | **69610** |
|  | NA × (WL vs. S) × Experiment | 0.012 | 0.026 | -0.038 | 0.062 | 65325 |
|  |  |  |  |  |  |  |
| *Group-level effects* | *Participant_sd (Intercept)* | 0.153 | 0.012 | 0.131 | 0.179 | 7187 |
|  | sd(Name Agreement) | 0.067 | 0.007 | 0.054 | 0.081 | 28946 |
|  | sd(Speech vs. Quiet) | 0.026 | 0.011 | 0.003 | 0.046 | 11714 |
|  | sd(Word List vs. Sentence) | 0.008 | 0.005 | 0 | 0.019 | 33445 |
|  | sd(NA × (S vs. Q)) | 0.02 | 0.014 | 0.001 | 0.054 | 24533 |
|  | sd(NA × (WL vs. S)) | 0.023 | 0.014 | 0.001 | 0.053 | 22589 |
|  | *Item_sd (Intercept)* | 0.041 | 0.022 | 0.002 | 0.074 | 1562 |
|  | sd(Name Agreement) | 0.083 | 0.044 | 0.004 | 0.147 | 1599 |
|  | sd(Speech vs. Quiet) | 0.139 | 0.054 | 0.023 | 0.225 | 2527 |
|  | sd(Word List vs. Sentence) | 0.112 | 0.044 | 0.018 | 0.182 | 2874 |
|  | sd(Experiment) | 0.018 | 0.009 | 0.001 | 0.035 | 7237 |
|  | sd(NA × (S vs. Q)) | 0.273 | 0.108 | 0.041 | 0.447 | 2380 |
|  | sd(NA × (WL vs. S)) | 0.226 | 0.087 | 0.039 | 0.365 | 2790 |
|  | sd(NA × Experiment) | 0.035 | 0.019 | 0.002 | 0.07 | 7414 |
|  | sd( (S vs. Q) × Experiment) | 0.041 | 0.023 | 0.002 | 0.084 | 6087 |
|  | sd( (WL vs. S) × Experiment) | 0.04 | 0.021 | 0.002 | 0.08 | 5466 |
|  | sd(NA × (S vs. Q) × Experiment) | 0.08 | 0.046 | 0.004 | 0.169 | 5992 |
|  | sd(NA × (WL vs. S) × Experiment) | 0.081 | 0.043 | 0.005 | 0.16 | 5395 |
|  | | | | | | |
| ***Log-transformed total pause time*** | | | | | | |
| *Population-level effects* | Intercept | 6.284 | 0.062 | 6.163 | 6.405 | 4174 |
|  | Name Agreement | **-0.589** | **0.055** | **-0.697** | **-0.481** | **26776** |
|  | Speech vs. Quiet | 0.031 | 0.072 | -0.111 | 0.174 | 37500 |
|  | Word List vs. Sentence | 0.037 | 0.06 | -0.083 | 0.155 | 37909 |
|  | Experiment | -0.03 | 0.113 | -0.252 | 0.19 | 3829 |
|  | NA × (S vs. Q) | 0.163 | 0.142 | -0.119 | 0.443 | 35595 |
|  | NA × (WL vs. S) | 0.017 | 0.121 | -0.219 | 0.255 | 37295 |
|  | NA × Experiment | 0.026 | 0.064 | -0.099 | 0.152 | 18480 |
|  | (S vs. Q) × Experiment | -0.045 | 0.059 | -0.162 | 0.071 | 51571 |
|  | (WL vs. S) × Experiment | -0.05 | 0.052 | -0.152 | 0.052 | 62542 |
|  | NA × (S vs. Q) × Experiment | **-0.234** | **0.112** | **-0.455** | **-0.012** | **63364** |
|  | NA × (WL vs. S) × Experiment | 0.037 | 0.106 | -0.17 | 0.246 | 59726 |
|  |  |  |  |  |  |  |
| *Group-level effects* | *Participant_sd (Intercept)* | 0.514 | 0.041 | 0.441 | 0.603 | 7707 |
|  | sd(Name Agreement) | 0.227 | 0.026 | 0.18 | 0.281 | 29906 |
|  | sd(Speech vs. Quiet) | 0.101 | 0.041 | 0.016 | 0.177 | 13912 |
|  | sd(Word List vs. Sentence) | 0.031 | 0.023 | 0.001 | 0.085 | 28697 |
|  | sd(NA × (S vs. Q)) | 0.112 | 0.073 | 0.005 | 0.27 | 16436 |
|  | sd(NA × (WL vs. S)) | 0.11 | 0.062 | 0.007 | 0.239 | 18382 |
|  | *Item_sd (Intercept)* | 0.118 | 0.06 | 0.006 | 0.205 | 1575 |
|  | sd(Name Agreement) | 0.217 | 0.123 | 0.01 | 0.406 | 1524 |
|  | sd(Speech vs. Quiet) | 0.348 | 0.141 | 0.052 | 0.576 | 2218 |
|  | sd(Word List vs. Sentence) | 0.289 | 0.124 | 0.031 | 0.487 | 2346 |
|  | sd(Experiment) | 0.058 | 0.034 | 0.003 | 0.125 | 8725 |
|  | sd(NA × (S vs. Q)) | 0.678 | 0.283 | 0.09 | 1.14 | 2238 |
|  | sd(NA × (WL vs. S)) | 0.575 | 0.248 | 0.067 | 0.97 | 2335 |
|  | sd(NA × Experiment) | 0.117 | 0.069 | 0.006 | 0.253 | 8968 |
|  | sd( (S vs. Q) × Experiment) | 0.153 | 0.085 | 0.009 | 0.318 | 6683 |
|  | sd( (WL vs. S) × Experiment) | 0.16 | 0.089 | 0.009 | 0.328 | 6183 |
|  | sd(NA × (S vs. Q) × Experiment) | 0.292 | 0.17 | 0.015 | 0.628 | 6590 |
|  | sd(NA × (WL vs. S) × Experiment) | 0.322 | 0.178 | 0.018 | 0.656 | 6527 |
|  | | | | | | |
| ***Log-transformed articulation time*** | | | | | | |
| *Population-level effects* | Intercept | 7.757 | 0.015 | 7.727 | 7.786 | 4999 |
|  | Name Agreement | **-0.089** | **0.019** | **-0.127** | **-0.052** | **37001** |
|  | Speech vs. Quiet | **0.046** | **0.014** | **0.018** | **0.074** | **49698** |
|  | Word List vs. Sentence | -0.005 | 0.012 | -0.029 | 0.019 | 45323 |
|  | Experiment | 0.025 | 0.024 | -0.021 | 0.073 | 3748 |
|  | NA × (S vs. Q) | 0.01 | 0.028 | -0.045 | 0.064 | 48524 |
|  | NA × (WL vs. S) | -0.002 | 0.024 | -0.049 | 0.046 | 47017 |
|  | NA × Experiment | 0.008 | 0.013 | -0.017 | 0.033 | 18403 |
|  | (S vs. Q) × Experiment | -0.016 | 0.009 | -0.034 | 0.003 | 52214 |
|  | (WL vs. S) × Experiment | -0.004 | 0.006 | -0.016 | 0.008 | 72990 |
|  | NA × (S vs. Q) × Experiment | -0.002 | 0.014 | -0.03 | 0.026 | 89838 |
|  | NA × (WL vs. S) × Experiment | -0.004 | 0.013 | -0.028 | 0.021 | 88482 |
|  |  |  |  |  |  |  |
| *Group-level effects* | *Participant_sd (Intercept)* | 0.11 | 0.009 | 0.095 | 0.13 | 8141 |
|  | sd(Name Agreement) | 0.053 | 0.005 | 0.044 | 0.063 | 21399 |
|  | sd(Speech vs. Quiet) | 0.03 | 0.005 | 0.021 | 0.041 | 29762 |
|  | sd(Word List vs. Sentence) | 0.007 | 0.004 | 0 | 0.015 | 26055 |
|  | sd(NA × (S vs. Q)) | 0.018 | 0.011 | 0.001 | 0.042 | 16427 |
|  | sd(NA × (WL vs. S)) | 0.014 | 0.01 | 0.001 | 0.036 | 16253 |
|  | *Item_sd (Intercept)* | 0.043 | 0.024 | 0.002 | 0.077 | 1422 |
|  | sd(Name Agreement) | 0.086 | 0.048 | 0.004 | 0.154 | 1456 |
|  | sd(Speech vs. Quiet) | 0.064 | 0.036 | 0.003 | 0.117 | 1607 |
|  | sd(Word List vs. Sentence) | 0.056 | 0.03 | 0.003 | 0.102 | 1895 |
|  | sd(Experiment) | 0.008 | 0.005 | 0 | 0.017 | 12710 |
|  | sd(NA × (S vs. Q)) | 0.13 | 0.073 | 0.006 | 0.235 | 1537 |
|  | sd(NA × (WL vs. S)) | 0.116 | 0.061 | 0.006 | 0.205 | 1857 |
|  | sd(NA × Experiment) | 0.016 | 0.009 | 0.001 | 0.034 | 14920 |
|  | sd( (S vs. Q) × Experiment) | 0.01 | 0.007 | 0 | 0.028 | 33328 |
|  | sd( (WL vs. S) × Experiment) | 0.01 | 0.007 | 0 | 0.027 | 25544 |
|  | sd(NA × (S vs. Q) × Experiment) | 0.02 | 0.015 | 0.001 | 0.056 | 30810 |
|  | sd(NA × (WL vs. S) × Experiment) | 0.02 | 0.014 | 0.001 | 0.054 | 26730 |

*Note.* NA refers to name agreement, WL refers to word lists, S refers to sentences, and Exp refers to Experiment.
